# Longitudinal Evaluation of Gut Bacteriomes and Viromes after Fecal Microbiota Transplantation for Eradication of Carbapenem-Resistant *Enterobacteriaceae*

**DOI:** 10.1128/msystems.01510-21

**Published:** 2022-06-01

**Authors:** Qin Liu, Tao Zuo, Wenqi Lu, Yun Kit Yeoh, Qi Su, Zhilu Xu, Whitney Tang, Keli Yang, Fen Zhang, Louis H. S. Lau, Rashid N. S. Lui, Miu Ling Chin, Rity Wong, Chun Pan Cheung, Wenyi Zhu, Paul K. S. Chan, Francis K. L. Chan, Grace C. Lui, Siew C. Ng

**Affiliations:** a Center for Gut Microbiota Research, The Chinese University of Hong Kong, Hong Kong, China; b Department of Medicine and Therapeutics, The Chinese University of Hong Kong, Hong Kong, China; c Microbiota I-Center, Hong Kong, China; d Institute of Digestive Disease, State Key Laboratory of Digestive Disease, LKS Institute of Health Sciences, The Chinese University of Hong Kong, Hong Kong, China; e Department of Microbiology, The Chinese University of Hong Kong, Hong Kong, China; Mayo Clinic

**Keywords:** CRE, FMT, bacteriome, virome

## Abstract

Understanding the role of fecal microbiota transplantation (FMT) in the decolonization of multidrug-resistant organisms (MDRO) is critical. Specifically, little is known about virome changes in MDRO-infected subjects treated with FMT. Using shotgun metagenomic sequencing, we characterized longitudinal dynamics of the gut virome and bacteriome in three recipients who successfully decolonized carbapenem-resistant *Enterobacteriaceae* (CRE), including Klebsiella spp. and Escherichia coli, after FMT. We observed large shifts of the fecal bacterial microbiota resembling a donor-like community after transfer of a fecal microbiota dominated by the genus *Ruminococcus*. We found a substantial expansion of Klebsiella phages after FMT with a concordant decrease of Klebsiella spp. and striking increase of Escherichia phages in CRE E. coli carriers after FMT. We also observed the CRE elimination and similar evolution of Klebsiella phage in mice, which may play a role in the collapse of the Klebsiella population after FMT. In summary, our pilot study documented bacteriome and virome alterations after FMT which mediate many of the effects of FMT on the gut microbiome community.

**IMPORTANCE** Fecal microbiota transplantation (FMT) is an effective treatment for multidrug-resistant organisms; however, introducing a complex mixture of microbes also has unknown consequences for landscape features of gut microbiome. We sought to understand bacteriome and virome alterations in patients undergoing FMT to treat infection with carbapenem-resistant *Enterobacteriaceae*. This finding indicates that transkingdom interactions between the virome and bacteriome communities may have evolved in part to support efficient FMT for treating CRE.

## INTRODUCTION

Drug-resistant enteric bacteria, including carbapenem-resistant *Enterobacteriaceae* (CRE) and vancomycin-resistant enterococci (VRE), are emerging worldwide (1, 2). Intestinal colonization by multidrug-resistant organisms (MDRO) may predispose at-risk individuals to invasive infection, which is associated with high mortality and can serve as a reservoir for spreading the bacteria to other patients (3). Within-patient transfer of high-risk carbapenemase-encoding plasmids has been reported via intestinal microbiota of colonized patients and may contribute to the overall dissemination of carbapenem resistance in the clinical setting (4), giving rise to an urgent need for effective and safe strategies to prevent and eradicate intestinal MDRO colonization (5).

Fecal microbiota transplantation (FMT) is highly effective in the treatment of recurrent Clostridioides difficile infections (CDI) (6) and has recently emerged as a promising therapy for decolonization of intestinal multidrug-resistant microorganisms (7). In four small case series with various study protocols, FMT resulted in 33 to 50% decolonization of CRE (7–10). Previous studies revealed compositional alterations of the intestinal bacteriome and the donor-derived engraftment as assessed by fecal sampling before and after FMT (11). FMT would alter recipient microbial ecology in carriers of carbapenem-resistant *Enterobacteriaceae* (12). However, the fate of native and introduced microbes may not be clearly associated with the success of FMT, and the mechanisms by which species are enriched or cleared after FMT in recipients remain unclear (9). Apart from the bacterial community, accumulating evidence suggest that gut viruses are also associated with FMT treatment outcomes in FMT (13, 14).

To date, there are limited data on how FMT affects CRE carriage in relation to the gut microbiome after FMT. Using shotgun metagenomic sequencing, we report for the first time comprehensive longitudinal dynamics of the gut bacteriome and virome in three CRE-positive patients who successfully decolonized CRE following FMT.

## RESULTS

### Intestinal CRE was eradicated after FMT.

**FIG 1:**
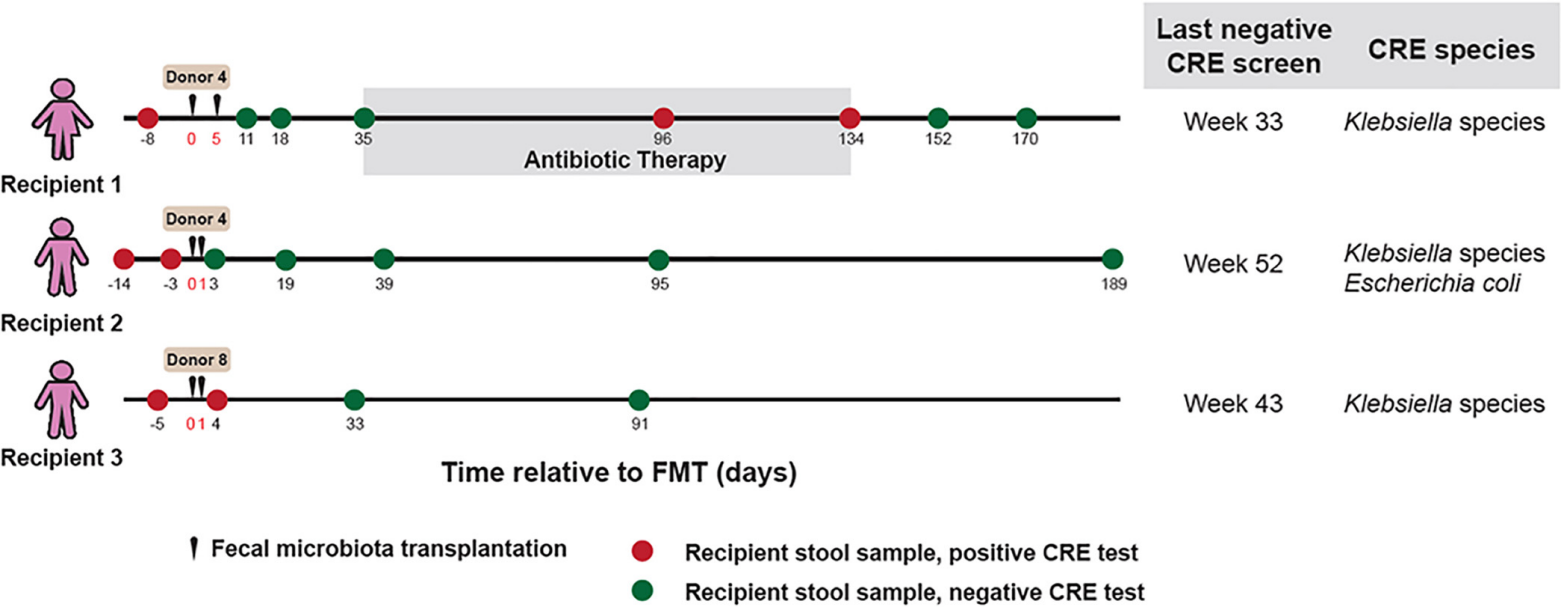
FMT to treat CRE. Timeline of sample collection for donors and CRE recipients. Results for CRE were based on rectal swabs from the recipients.

Three CRE-positive patients showed persistently positive isolation of carbapenem-resistant Klebsiella species and Escherichia coli from two consecutive rectal swabs at least 1 week apart. They received two FMTs and successfully cleared the CRE (Fig. 1A). Recipient 1 (female, 90 years old) underwent two FMTs 5 days apart. Tests for CRE performed at 11, 18, and 35 days after the first FMT were negative. Recipient 1 then developed infection of a foot ulcer and received antibiotic therapy (four courses of amoxicillin-clavulanic acid [Augmentin]) from day 36 to 134 (week 6 to week 19) after the first FMT and tested positive for CRE on days 96 and 134. However, the patient became CRE negative on days 152 and 170 after completion of antibiotic therapy. Recipient 2 (male, 70 years old) and recipient 3 (male, 74 years old) each received two FMTs on consecutive days and tested negative for CRE on days 3 and 33, respectively, after FMT.

### FMT induced significant changes in bacteriome diversity and composition.

**FIG 2:**
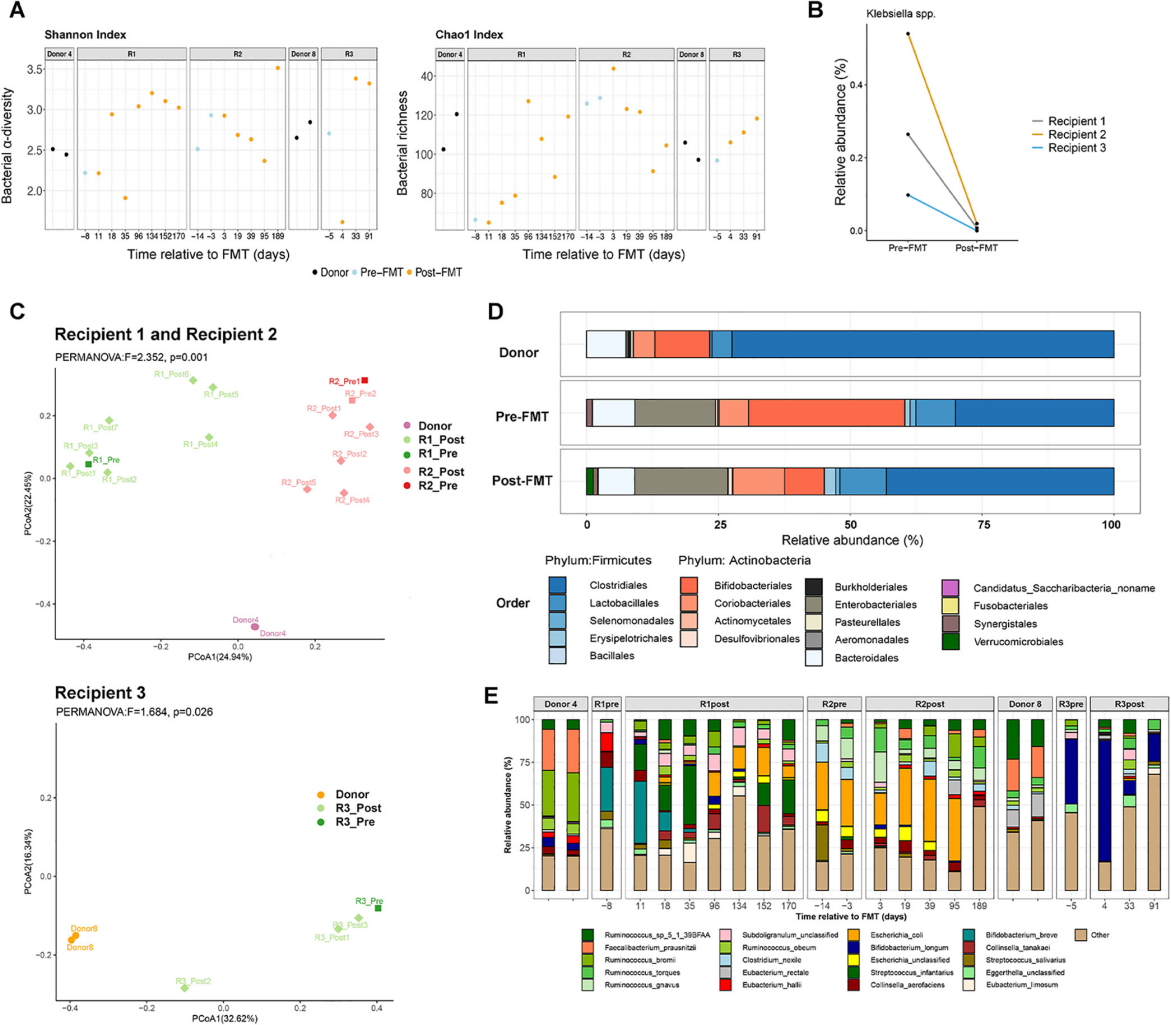
Analysis of bacterial composition of FMT donors and recipients. (A) Alpha diversity (Shannon diversity) and richness (Chao1 index) of fecal bacterial composition of donors, recipients before FMT, and recipients after FMT at different time points. (B) Relative abundance of Klebsiella spp. detected in all recipients substantially decreased after FMT. (C) PCoA based on Bray-Curtis distance for recipients and donors. PCoA plot showing separation of relative abundance of gut microbiota populations before and after FMT. (D) Fecal bacterial composition profile at the order level in donors and in CRE-colonized FMT recipients pre-FMT and post-FMT. Bacterial community composition changed toward that of donors, with increased *Firmicutes* and decreased *Actinobacteria* in recipients 2 weeks after FMT. The dominance of *Firmicutes* in stool of donors and CRE recipients post-FMT is denoted by the blue color. (E) Relative abundance of fecal bacterial taxa (at species level) of the donors and recipients.

We next assessed alteration in the fecal bacteriome in CRE-infected subjects after FMT. Fecal samples were collected serially every 4 weeks up to 24 weeks (recipients 1 and 2) or 12 weeks (recipient 3). Although alpha diversity of the recipients’ fecal bacteriomes fluctuated over time, all recipients exhibited increased fecal bacterial diversity and richness at the last follow-up after FMT compared to their corresponding baseline levels (Fig. 2A; Fig. S1A). Following FMT, the relative abundance of Klebsiella spp. (CRE species) detected in all recipients and E. coli in recipient 2 (CRE E. coli carrier) substantially decreased (Fig. 2B; Fig. S1B), which was further corroborated by qPCR quantification. Next, we compared species-level dissimilarity (using Bray-Curtis dissimilarity) of stool bacterial community composition in the three CRE subjects before and after FMT with that of their respective donors. Principal-coordinate analysis (PCoA) with Bray-Curtis dissimilarity demonstrated that the gut bacteriome in CRE subjects resembled that of the donor immediately after FMT and remained significantly different from the CRE subjects’ own compositions before FMT (Fig. 2C). Recipient 1 developed infection of a foot ulcer, received antibiotic therapy (four courses of amoxicillin-clavulanic acid [Augmentin]) from day 36 to 134 (week 6 to week 19) after the first FMT, and tested positive for CRE on days 96 and 134. This may have led to the observation that R1_post5 and R1_post6 were more different from the donor than R1_post1.

10.1128/msystems.01510-21.3 FIG S1 PCoA and community profile of total bacterial communities in donors and recipients before and after FMT. (A) PCoA based on Bray-Curtis dissimilarity for recipients before and after FMT at different time points. (B) Relative abundance of Escherichia coli detected in recipient 2 substantially decreased after FMT. (C) Proportion of the donor source in three recipients’ samples using SourceTracker. (D) Gut bacterial composition profile at the order level in the stool of donors and CRE recipients before and after FMT. (E) Venn diagram showing the number of species in stool of donors and CRE recipients before and after FMT. (F) LEfSe comparing bacterial composition in pre-FMT (green) and post-FMT (red) samples. Represented are all taxa that were significantly distinct with LDA scores of >2.0. Download FIG S1, JPG file, 0.4 MB. Copyright © 2022 Liu et al. 2022 Liu et al. https://creativecommons.org/licenses/by/4.0/ This content is distributed under the terms of the Creative Commons Attribution 4.0 International license.

To explore transfer of donor-derived microbial taxa to recipients, we applied Bayesian source tracking (15) to compare bacterial composition between FMT recipients and their corresponding donors. We observed distinct microbial engraftment ratios in the three recipients. Engraftment of donor-derived bacteria was more pronounced when FMT was performed on consecutive days (in recipient 2 and recipient 3, mean engraftment ratios were 37.2% and 13.3%, respectively) than when FMTs were performed 5 days apart (in recipient 1, the mean engraftment ratio was 2.8%) (Fig. S1C). These data suggest that consecutive FMT infusions may facilitate better engraftment of donor-derived microorganisms in the recipient.

We found a marked shift in bacterial community composition toward that of the donor, with increased *Firmicutes* and decreased *Actinobacteria*, in recipients 2 weeks after FMT (Fig. 2D and E; Fig. S1D and S2). At the species level, members of the *Clostridiales*, such as *Ruminococcus* sp. strain 5_1_39BFAA, were obviously engrafted in recipients (Fig. 2E), as demonstrated by their predominance in donors’ and recipients’ bacteriomes after FMT. The proportions of *Ruminococcus* increased significantly after FMT (*P* < 0.05, Wilcoxon test) (Fig. S1F). Differential analysis by linear discriminant analysis (LDA) effect size (LEfSe) between pre-FMT and post-FMT samples pinpointed the enrichment of *Ruminococcus* sp. strain 5_1_39BFAA after FMT in CRE recipients (Fig. S1F) (false discovery rate [FDR]-corrected *P* value < 0.05; LDA effect size = 4.3). Recipients and donors shared a large number of bacterial species (142 species) in their fecal bacteriomes before FMT. After FMT, recipients shared an additional 27 species with the donors (Fig. S1E). These donor-like assemblages and features of donor-transferred taxa after FMT suggest engraftment of donor microbiomes in recipients and a potential role of bacterium-bacterium competitive exclusion dominated by *Ruminococcus* for CRE decolonization.

10.1128/msystems.01510-21.4 FIG S2 Comparison of the relative abundance of the bacterial taxa between samples obtained from donors, recipients before FMT, and recipients after FMT. A Wilcoxon test was performed to analyze the differences. Download FIG S2, JPG file, 0.1 MB. Copyright © 2022 Liu et al. 2022 Liu et al. https://creativecommons.org/licenses/by/4.0/ This content is distributed under the terms of the Creative Commons Attribution 4.0 International license.

### Functional alterations of the bacteriome after FMT.

**FIG 3:**
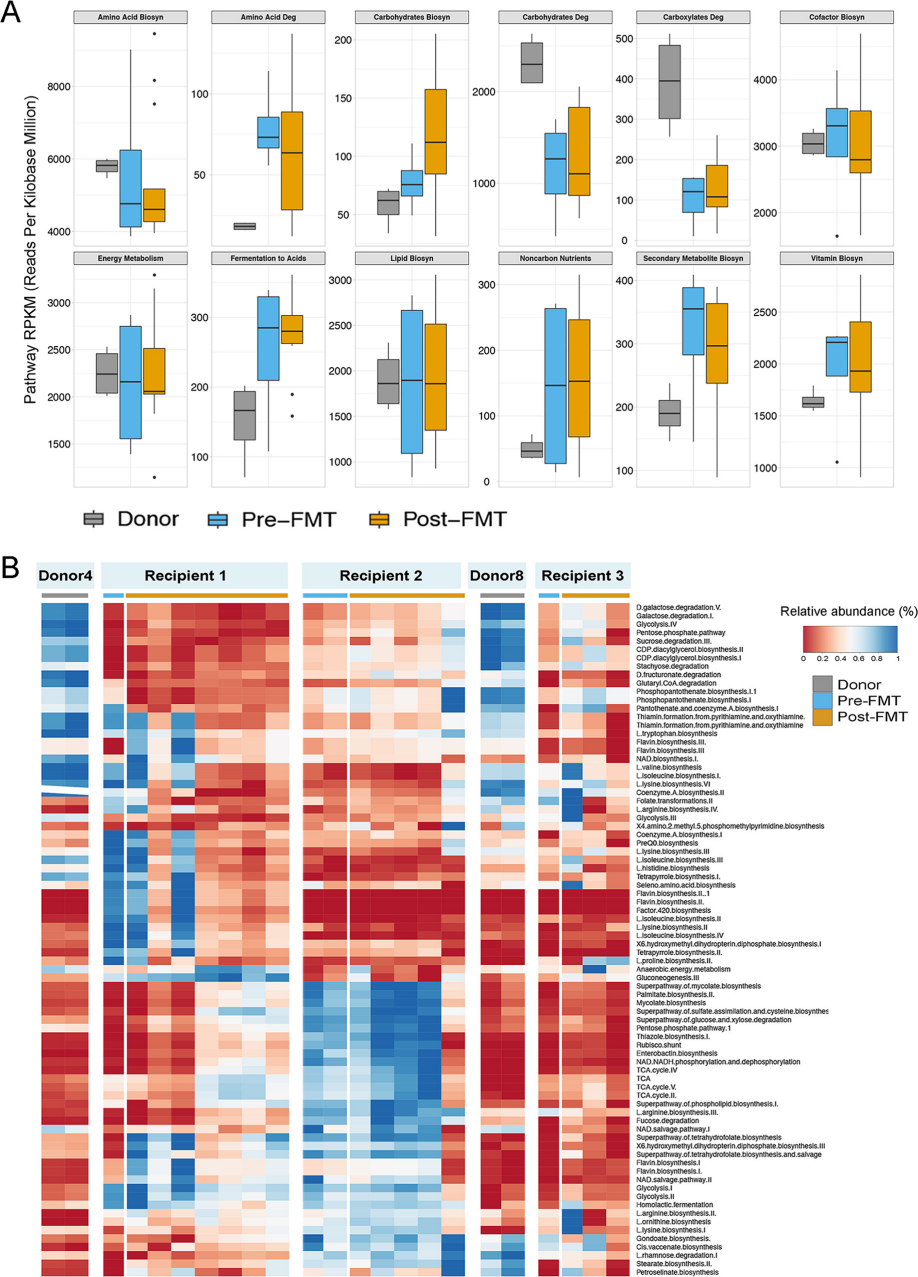
Functionality alterations following FMT in donor and recipients. (A) Metagenomic functional predictions for donor, pre-FMT, and post-FMT samples. Mean relative gene pathway abundances for pre- and post-FMT samples were not significantly different (Mann-Whitney test; *P* > 0.05). Gene pathway abundances were calculated using the HUMAnN pipeline and grouped by major functional categories. (B) Abundance distribution of bacteria function pathways in donors and in recipients following FMT.

We next investigated the functionality of the bacteriomes of recipients following treatment with FMT. Total bulk DNA sequencing reads from the metagenomic data set were evaluated against reference databases (MetaCyc) to interrogate the abundance of various metabolic pathways. We found that two prominent pathways in donors, the pentose phosphate pathway (nonoxidative branch) and the UDP-*N*-acetylmuramoyl-pentapeptide biosynthesis pathway (Fig. S3A and B), were significantly more abundant than in pre-FMT samples (*P* < 0.05). Notably, the differential presences of these two pathways between CRE carriers and donors were primarily contributed by the bacterial genera *Faecalibacterium* and *Ruminococcus* (Fig. S3C and D), both of which are members of the family *Ruminococcaceae*, implying a bacteriome function-level alteration along with taxonomic-level alteration in CRE. We identified marked differences in overall predicted microbiome functionality between the donors and pre-FMT recipients; however, these differences were not reflected in recipients post-FMT (Fig. 3A). In recipients, there were no significant changes in representative functionality before and after FMT. Though the bacteriome functionality profiles of the two donors were similar, the post-FMT functionalities of the recipients were considerably different from each other (Fig. 3B). For instance, recipient 2 showed greater abundances of tricarboxylic acid cycle (TCA)-related functions and lower abundances of lysine and isoleucine biosyntheses than recipients 1 and 3. The core functionality remained stable with respect to each study subject over the course of post-FMT follow-up. However, the persistently salient discrepancies in microbiome functionality between these recipients suggest a significant interpersonal variation among individuals after FMT treatment.

10.1128/msystems.01510-21.5 FIG S3 Relative abundance of the two most prominent pathways that were significantly more abundant in donors: the pentose phosphate pathway (nonoxidative branch) (a) and UDP-*N*-acetylmuramoyl-pentapeptide biosynthesis (b). Taxonomic contribution to the pentose phosphate pathway (nonoxidative branch) (c) and UDP-*N*-acetylmuramoyl-pentapeptide biosynthesis (d). Download FIG S3, JPG file, 0.10 MB. Copyright © 2022 Liu et al. 2022 Liu et al. https://creativecommons.org/licenses/by/4.0/ This content is distributed under the terms of the Creative Commons Attribution 4.0 International license.

### Characterization of the gut virome in patients after FMT.

**FIG 4:**
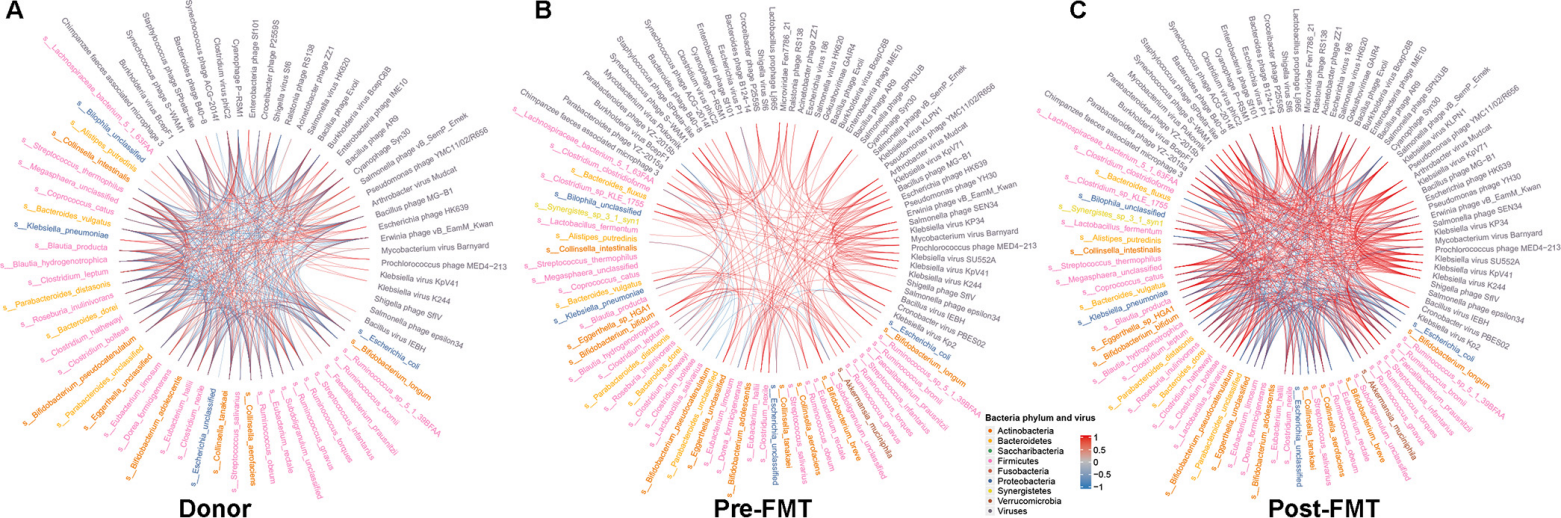
FMT influences gut microbiome interactions in patients with CRE. The correlation networks of the gut bacteriomes and gut viromes of patients with CRE before and after FMT (last collection sample from each recipient) and donors. Vertices indicate omics variables, and lines indicate a significant Pearson correlation coefficient at a |ρ| value of >0.6 and a *P* value of <0.05.

**FIG 5:**
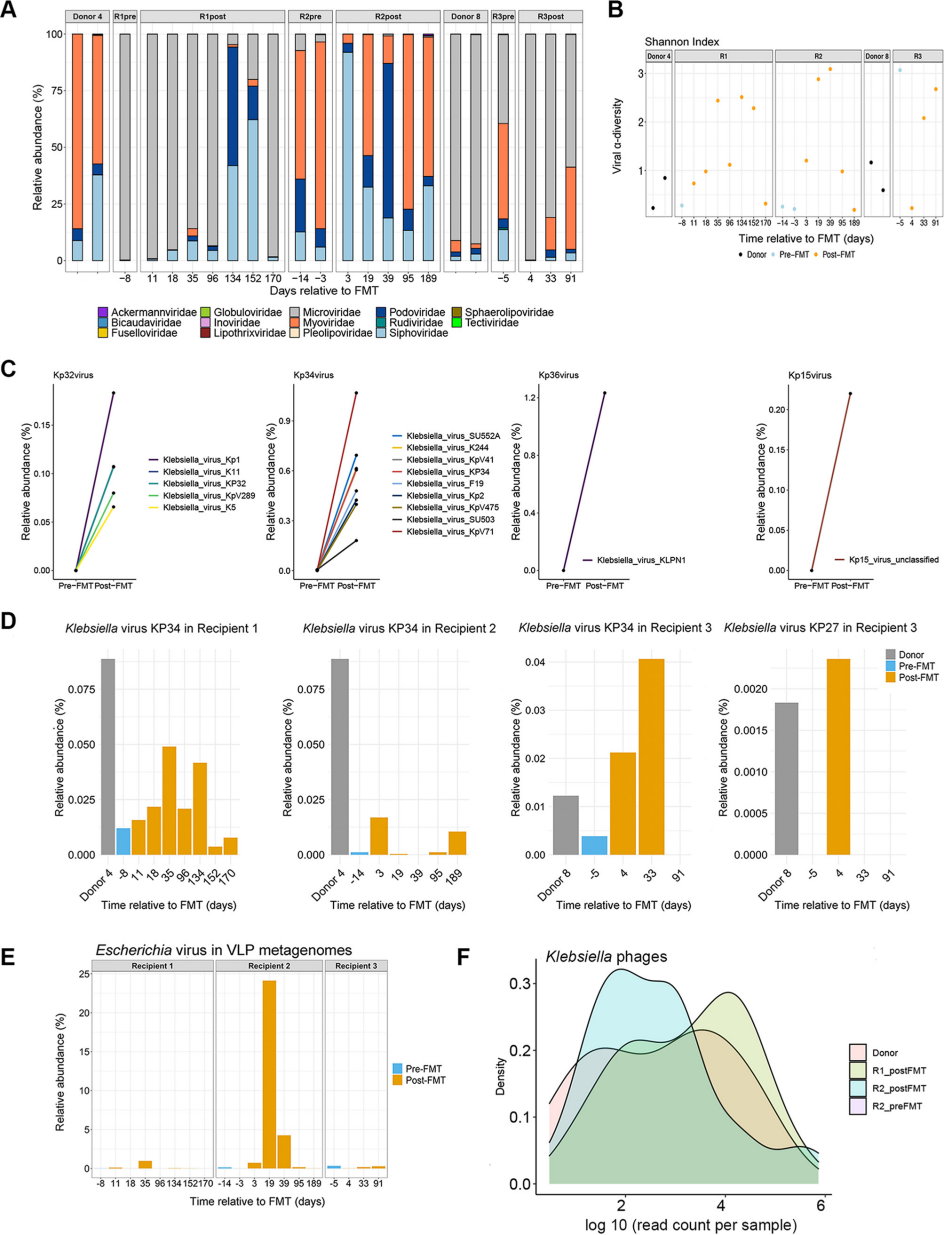
Analysis of viral composition of donors and CRE recipients, and alterations of bacteriophages following FMT. (A) Relative abundance of gut virome at the order level of donor and recipient before and after FMT. (B) Alpha diversity (Shannon’s diversity) of viromes in stools of donors and recipients at different time points after FMT. (C) Relative abundance of Klebsiella species in recipients (samples from recipient 1 prior to starting amoxicillin-clavulanic acid [Augmentin]). (D) Alterations of Klebsiella phages relative abundance from stool VLP metagenomes before and after FMT. (E) Alteration of Escherichia phages stool VLP metagenomes from three recipients before and after FMT. (F) Density plots of log_10_ (read count per sample) distribution of Klebsiella phages.

To explore the links between gut bacteriome and the virome, we carried out interaction correlation analysis of the relative abundance of gut microbiomes. Tight connections were identified in both donor and post-FMT recipient microbiomes (Fig. 4A and C). However, the correlation network was clearly different from that of donor and post-FMT microbiomes, as only ~35% of correlations were common compared to donors. Nevertheless, correlations between bacteriome and virome were enriched in the two groups, as regards direction (positive or negative), supporting the finding that FMT impacts the transkingdom interactions of gut microbiome components. We next analyzed the bacteriophages in the donors’ and the recipients’ fecal samples. Before FMT, the proportions of *Myoviridae* and *Microviridae* were high in terms of relative abundance (Fig. 5A). Concomitantly, we found a striking increase in Klebsiella phages in all recipients (Fig. S4B), including Kp32virus (T7-like phage), Kp34virus (ϕKMV-like phage), Kp36virus (*Siphoviridae* phage), and Kp15virus (*Myoviridae* phages) after FMT (Fig. 5C) by metagenomic data analysis. Among these species, Kp34virus was previously reported as a novel virus belonging to the subfamily *Autographivirinae* and lytic for extended-spectrum-β-lactamase-producing Klebsiella pneumoniae strains (16). These findings indicate a potential mechanism through which FMT could reduce Klebsiella spp. in the gut via transfer or expansion of bacteriophages. Recipient 2 also carried CRE E. coli apart from CRE Klebsiella spp. before FMT (Fig. 1). By examining the relative abundance of Escherichia phages in all three recipients, we found a marked increase of Escherichia phage only in recipient 2 (CRE E. coli carrier) after FMT (Fig. 5E), supporting targeted bacteriophage expansion for CRE causative bacteria in FMT.

10.1128/msystems.01510-21.6 FIG S4 Alpha diversity of viral populations and bacteriophages changes before and after FMT. (A) K. pneumoniae qPCR relative levels in stools. Related to Fig. 3A. (B) Alteration of total relative abundance of Klebsiella phages from VLP-derived metagenomes in three recipients. (C) Relative abundance of Klebsiella phages from VLP-derived metagenomes. (D) Relative abundance of Escherichia phages from VLP-derived metagenomes. Download FIG S4, JPG file, 0.2 MB. Copyright © 2022 Liu et al. 2022 Liu et al. https://creativecommons.org/licenses/by/4.0/ This content is distributed under the terms of the Creative Commons Attribution 4.0 International license.

Using virus-like particle (VLP) sequencing, we found that most Klebsiella phages and Escherichia phages were present at low abundance or absent in stools of either donors or recipients prior to FMT (Fig. S4C and D). As metagenomic analysis on the VLP enriched virome data set provide information only on the presence of actively replicating phage particles at the time of sampling (17), we harnessed our whole-community metagenomic data set, which contained significant fractions of sequence data, to identify the origin of these expanded phages predating CRE bacteria. We found the presence of donor-derived Kp34virus and Klebsiella virus KP27 in recipients’ bulk metagenomes (Fig. 5D). Furthermore, we assembled bulk metagenomic sequence reads into contigs and screened with the gene enrichment-based method VirSorter (18). The viral contigs predicted as category 1 and 2 (intact and incomplete virus, respectively) were mapped back onto randomly subsampled (approximately 6 million reads per sample) sequences. The predicted open reading frames from these viral contigs were mapped to the viral protein database in viral RefSeq protein (v84). We identified 86 Klebsiella phage contigs with a mean of 59,422 ± 173,404 counts per sample. Numbers of mapped sequences that could be assigned to Klebsiella phage were 7,146, 1.5, and 27,425 (mean value) in donor, pre-FMT, and post-FMT microbiotas of all subsampled reads (Fig. 5F). The marked higher abundance level in donors also implied that the bloom of bacteriophages after FMT were more likely of donor origin.

### FMT decolonizes carbapenem-resistant Klebsiella pneumoniae and reconstitutes the microbiota in mice.

**FIG 6:**
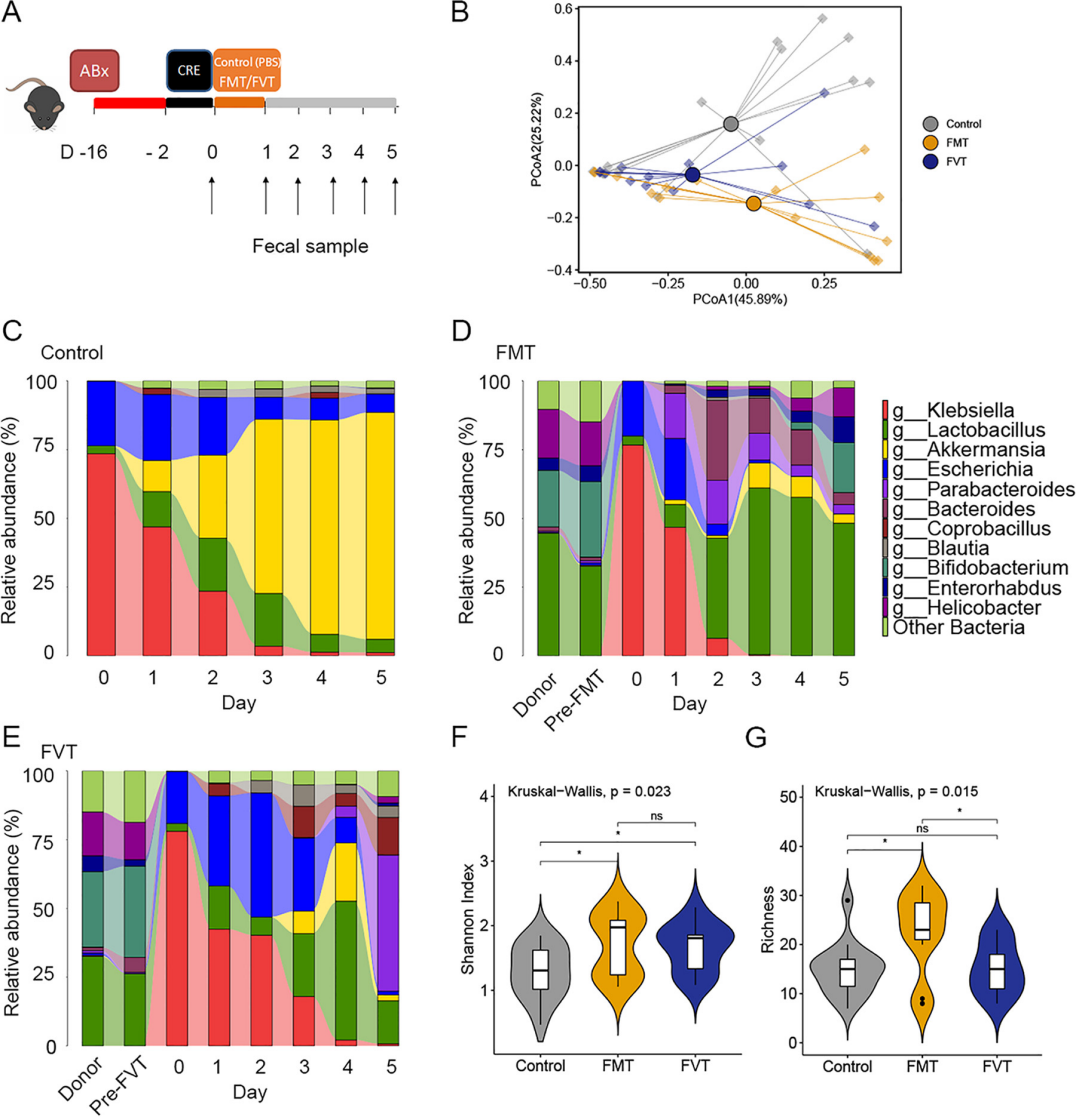
FMT decolonizes carbapenem-resistant Klebsiella pneumoniae and reconstitutes the microbiota in mice. (A) Experimental scheme for Klebsiella pneumoniae challenge and treatment with FMT and with FVT alone. (B) PCoA of gut microbiota composition of mice stool identifies separation between control (gray), FMT (yellow), and FVT (blue) samples. (C to E) Fecal microbiota composition at the genus level of treated mice (5 mice per group). (F and G) Diversity (Shannon index) and richness of mouse gut bacteriomes.

The data above indicated that FMT effectively eradicates CRE in recipients; hence, we performed experiments with CRE-challenged mice, focusing on virome and bacteriome alteration after FMT. We tested whether the FMT or fecal virome fraction transplantation alone (FVT; actively replicating phage particles, lytic phage) could clear CRE from the gut of densely colonized mice (Fig. 6A). CRE eradication was observed in a parallel *in vivo* study of mice specifically treated by gavage with carbapenem-resistant Klebsiella pneumoniae isolated from CRE patients. Carbapenem-resistant K. pneumoniae was introduced into antibiotic-treated mice, and the mice were treated with fecal microbiota/virome fractions from healthy mice twice by oral gavage. All mice were densely colonized with CRE prior to the treatment. We observed three distinct clusters in mice treated by FMT and FVT, indicating significantly distinct microbiota community compositions (Fig. 6B) (*P *< 0.05). We confirmed the clearance by the culture method (stool suspension plated on chromID C S; bioMérieux, France). By the culture method, we found that both FMT and FVT appear to clear CRE: FMT resulted in clearance on day 3, while FVT resulted in clearance on day 10, whereas controls had persistently positive isolation of CRE by the end of experiment, at least 1 month after challenge.

**FIG 7:**
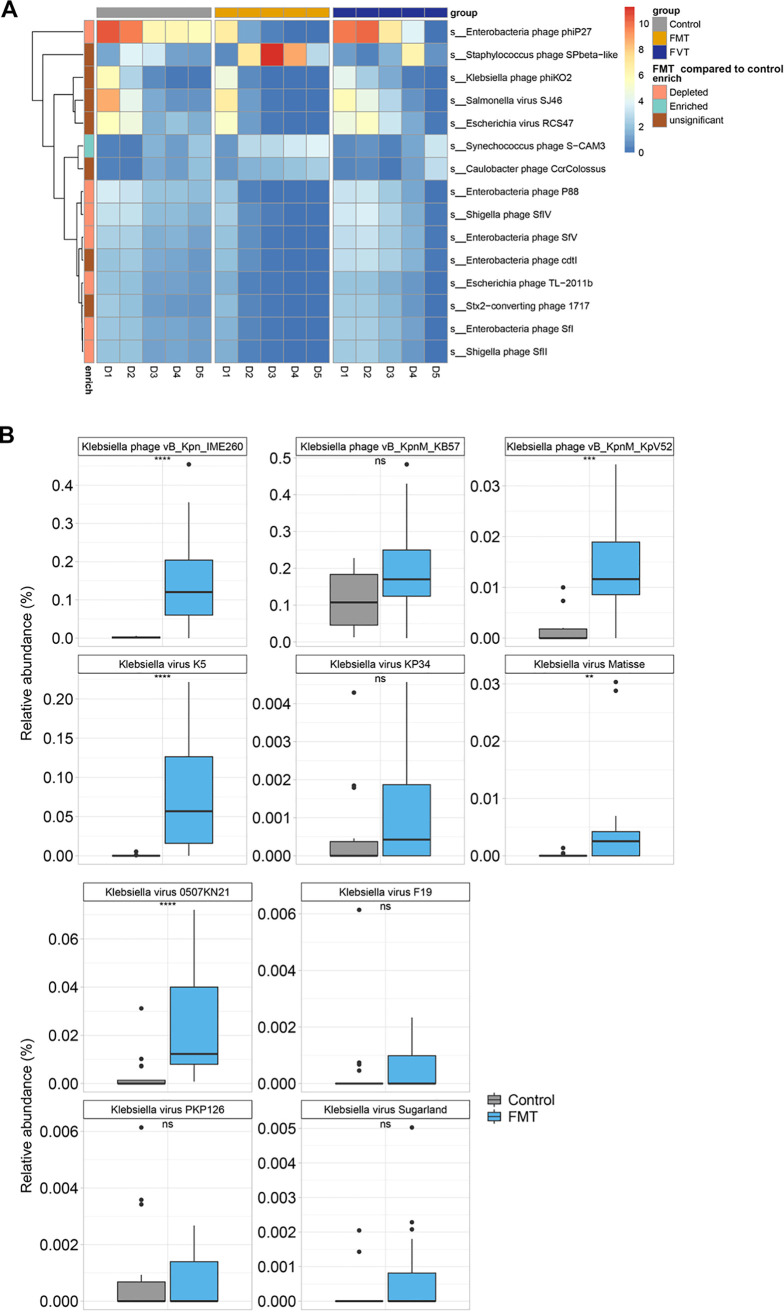
Alteration of mice gut virome and impact of FMT in CRE-challenged mice. (A) Abundance distribution of virus in mouse stool following FMT. (B) The relative abundance of 10 predominant Klebsiella phages increased after FMT.

Using metagenomic sequencing, we first characterized the fecal microbiota taxonomic composition. Taxonomic analysis demonstrated that Klebsiella and Escherichia were highly represented on day 0, approximately 75% and 20%, respectively (Fig. 6C to E). While the microbiota composition of control mice was *Akkermansia* dominated, FMT/FVT resulted in a highly diverse population on day 5 (Fig. 6D and E). Administration of healthy fecal microbiota to K. pneumoniae-challenged mice resulted in engraftment and restoration of normal gut microbial community structure. Importantly, the FMT resulted in progressive reduction in Klebsiella sp. levels and achieved clearance on day 3 (Fig. 6D). Conversely, at day 5, the genus Klebsiella still represented approximately 4.18% and 0.78% of bacterial taxa in control and fecal virome recipient mice (Fig. 6C and E). We found that FMT-treated mice had bacterial compositions more similar to that of the donor than FVT-treated mice at day 5. Despite these differences, fecal microbial communities after FMT or FVT were similarly diverse, as quantified by the Shannon index (Fig. 6F). Bacterial diversity (Shannon index) and richness after FMT were significantly greater than those in control mice (*P* < 0.05) (Fig. 6F and G). A heat map displays the trajectory of gut virus derived from mouse stool VLP metagenomes (Fig. 7A). At day 1, *Enterobacteria* phage phiP27, Klebsiella phage phiKO2, Salmonella virus SJ46, and Escherichia virus RCS47 were more prominent. Notably, we also observed higher relative abundances of 10 Klebsiella phages in samples from FMT-treated mice than in control samples (Fig. 7B).

## DISCUSSION

Emerging evidence suggests that FMT has the potential to reduce colonization of multidrug-resistant organisms (MDRO) in the human gut, but the underlying mechanism remains unknown, although it is likely to be similar to that of eradication of C. difficile, i.e., outcompetition of MDRO and repopulation with a normal gut microflora (19). Competition between microorganisms is not limited to access for nutritional resources. Production of bacteriocins such as nisin and lacticin 3147, which are antimicrobial peptides with bactericidal or bacteriostatic activity, is a more active strategy against competitors (20, 21). FMT outcome would be a synergistic effect of bacterium-virome interaction and outcompetition by bacteria introduced by FMT. In this proof-of-concept study, we observed substantial decreases in the relative abundances of Klebsiella spp. accompanied by a marked increase in Klebsiella phages in three recipients who successfully cleared CRE after FMT. These data highlight for the first time the intimate interplay of the predator-prey relationship between phage and the bacteria in the human gut during FMT treatment for multidrug-resistant microorganisms. We also report an increase of Escherichia phage in a CRE E. coli carrier after FMT, which further supports the importance of bacteriophages for clearing specific CRE bacteria.

Unlike targeted microbial therapies and specific bacteriophage therapy, which have a target in the host, FMT is **“**nonselective” and alters the entire microbiota. Until recently, it has been unclear which components of donor stool drive FMT efficacy, i.e., whether it is bacteria, viruses, fungi, archaea, proteins, chemical mediators, or the interactions between these entities. Our data provide new and novel evidence that increased level of Klebsiella phages and Escherichia phages brought by FMT may play a key role in CRE decolonization. All patients were confirmed to have clearance of CRE following treatment with two FMTs. To date, there are only three reports on one-time FMT resulting in successful CRE decolonization; however, they had lower clearance success rates of 33% (9) (two of six patients) after 1 month and 50% (10) (four of eight patients) after 3 months, and 69.2% of participants who received consecutive days of FMT achieved eradication of CRE after 1 month (22). The successful outcome of CRE eradication in all subjects could be due to the protocol of two consecutive FMTs (10), indicating a need to optimize FMT strategies. Furthermore, we observed a greater donor-derived proportion of bacterial community in recipients given two consecutive-day FMTs than in those given a single FMT. Given the small sample size of our study, future large-scale studies on MDRO are warranted to investigate the mechanism of FMT in MDRO decolonization.

In addition, our serial sampling allows us to study the longitudinal dynamics of gut bacterial and viral communities and their interactions after FMT. Engraftment of microbiome after FMT, especially by members of the phylum *Firmicutes* and the family *Ruminococcaceae*, supports a more favorable microbiome profile and MDRO resolution in CRE recipients by FMT. Findings derived from previous studies (23, 24) also revealed that FMT patients had an enrichment of *Ruminococcaceae* upon immunotherapy treatment for recurrent hepatic encephalopathy (rHE) and anti-programmed cell death 1 protein (PD-1). In this study, we found interindividual variability of gut microbiome composition after FMT. Recipient factors such as genetics, immunity status, inflammatory status (25), and microbiota have crucial roles in gut microbiome restoration after FMT. Following FMT, donor bacterial strains cohabit with those of the recipient. The colonization success for a given strain may be greater if another representative of the same species is also present in the recipient before FMT (26).

Bacteriophages are of biomedical importance because of their ability to infect bacteria and transmit genes to their bacterial hosts, resulting in altered microbiota compositions, antibiotic resistance, and novel metabolic capacity in distinct microbial lineages (27, 28). In this study, although the overall virome composition fluctuated in the recipients after FMT, alterations in the transkingdom interactions between the virome and bacteriome communities revealed a role of FMT in the recipients’ gut microbiome. Reset of the bacteria-phage ecology after FMT indicated a “kill-the-winner” dynamic (29), whereby upregulation of Klebsiella phage was accompanied by decrease in abundance of Klebsiella spp. after FMT. These data highlight the importance of restoration of a balanced relationship between the bacterial microbiome and virome after FMT. As bacteriophages propagate via lytic or lysogenic infection of bacteria (30), bacteriophages play an important role in restricting and eliminating antibiotic-resistant organisms. The increased levels of Klebsiella phages and Escherichia phages introduced by FMT support a new mechanism of FMT and its potential therapeutic use to target decolonization of MDRO. Phages are ubiquitous in bacterium-rich environments, including the gut, and phages are highly strain specific, which potentially makes the direct modulation of the gut microbiota feasible (31). As such, predation by phages and bacterium-bacteriophage coevolution could contribute to the effective decolonization of CRE Klebsiella species and CRE E. coli in recipients by FMT.

Given the alarming increase in the frequency of multidrug-resistant bacterial infections globally, phage therapy is gaining increasing interest among medical researchers. However, phages infect bacterial hosts with a very narrow range, and bacteria can rapidly develop resistance to phages, suppressing their effectiveness (32, 33). Development of resistance is less likely when multiple different phages are used simultaneously in a cocktail. Moreover, it is challenging to obtain a set of phages that is effective against all variants of a given causative pathogen. FMT is akin to an overall community containing an “active ingredient” of bacteriophages, and thus, it can be used as a means to overcome the disadvantages of unknown target bacteria as well as the inevitable development of phage resistance. Our data highlight the possibility and importance of viral transfer for causative CRE bacteria species. This pilot study was not able to define the origin of all bacteriophages and the specific host-bacteriophage relationship due to existing challenges in the analysis of viral metagenomes (34). Nonetheless, our data will serve as a reference in FMT practice and research for eradication of other antibiotic-resistant microorganisms.

The limitations of this study are in keeping with the nature of the proof-of-concept study design. It remains an exploratory study with a small sample size, and validation is needed in an independent cohort. For this pilot analysis we included all consecutive cases of subjects who received FMT for CRE, and the first three cases had successful FMT. To date, we do not have any cases of CRE infection with unsuccessful FMT, but we believe that this is an important consideration for future analysis and comparison. With emerging evidence that the virome is associated with FMT outcome, longitudinal studies are needed to provide further information on mechanisms of the roles of gut microorganisms in the resolution of MDRO.

From our pilot study, we cannot clearly define the exact mechanism of FMT action. In particular, it cannot be determined whether the observed taxonomic shifts in the microbiota promoted the clearance of MDRO or rather reflect the resolution of disease with re-establishment of a health-associated microbiome after transfer of the active agents from the donor. However, our findings support one plausible explanation, that phage blooms induced by FMT may act on the community dynamics of gut microbiota, thereby leading to a resolution of CRE. This is supported by the findings that (i) CRE decolonization and bloom of phage targeting CRE were observed in both human recipients and a mouse model and (ii) annotation of quality-controlled viral contigs revealed that phage might have originally derived from sequenced donor genomes.

In conclusion, this study showed successful decolonization of CRE by FMT. Analysis of multikingdom fecal microbiomes created new research opportunities for understanding the feature of MDRO clearance in CRE carriage patients by FMT. This study is unique, as it demonstrates direct evidence of bacterial repopulation, particularly *Ruminococcaceae*, and an inverse relationship between bacteriophages and CRE in recipients. The decolonization of CRE species, accompanied by an expansion of Klebsiella phages and Escherichia phages, after FMT supports the idea that phage-directed predation of bacteria may lead to CRE eradication. Our results suggest that the gut virome represents an important yet understudied component of the gut microbiome. Evaluating the virome composition of donors and bacteriophage regulations during FMT may be crucial for enhancing the efficacy of FMT in the treatment of various diseases.

## MATERIALS AND METHODS

### Clinical study of FMT for eradication of CRE in humans.

Patients who were ≥18 years old, had two or more stools or rectal swabs positive for CRE (see the supplemental material) at least 1 week apart, and had not received antimicrobial therapy for at least 48 h prior to infusion of FMT were recruited to a clinical trial (NCT03479710). Study approval was provided by The Joint Chinese University of Hong Kong, New Territories East Cluster Clinical Research Ethics Committee (the Joint CUHK-NTEC CREC; CREC reference no. 2017.555). All relevant ethical regulations were followed. Patients consented to participating in the study and agreed to the findings of the research being published. Patients who had active infection with CRE or vancomycin-resistant *Enterococcus* (VRE) requiring antimicrobial therapy, pregnancy, active gastrointestinal tract infection, or inflammatory disorders, had undergone recent intra-abdominal surgery, had short gut syndrome, used medications which alter gastrointestinal motility were excluded. CRE colonization was defined as the presence of any *Enterobacteriaceae* with resistance to any of the carbapenems. CRE colonization was confirmed by specific selective medium (chromID Carba Smart; bioMérieux, France).

In this study, patients received two FMTs using frozen donor stool samples. FMT solution (100 mL; raw stool, 50 g) in 0.9% sterile saline was infused over 2 to 3 min into the distal duodenum or jejunum via esophagogastroduodenoscopy (OGD). Stool samples were collected from patients before and after FMT prospectively. Each recipient received FMT from the same single donor for the two FMTs. Donor 4 was the donor source for recipients 1 and 2, and donor 8 was the donor source for recipient 3. Stools for FMT infusion were obtained from donors recruited to Stool Biobank for the Faculty of Medicine, The Chinese University of Hong Kong. Donors were volunteers from the general population, including spouses or partners, first-degree relatives, other relatives, friends, and others who were known or unknown to the potential patients. Donors were required to fulfil a set of eligibility criteria and passed screening laboratory tests for infectious diseases, including infection with CRE and VRE. A total of four controls were recruited from the community through advertisement and from the endoscopy center at the Prince of Wales Hospital from among subjects who had a normal colonoscopy (stools collected before bowel preparation), and two donors served as healthy controls. Additional stool samples that were CRE positive from two patients who spontaneously recovered were collected for gut microbiome comparison. All samples from patients with CRE, donors, and controls were processed simultaneously.

### Microbiome analysis.

Microbiome analysis was performed on stool samples from three CRE recipients before and after FMT and on samples collected from donors. Approximately 100 mg fecal sample was prewashed with 1 mL double-distilled water (ddH_2_O) and pelleted by centrifugation at 13,000 × *g* for 1 min. The fecal pellet was resuspended in 800 μL Tris-EDTA (TE) buffer (pH 7.5), supplemented with 1.6 μL 2-mercaptoethanol and 500 U lyticase (Sigma), and incubated at 37°C for 60 min. The sample was then centrifuged at 13,000 × *g* for 2 min, and the supernatant was discarded. Fecal DNA was subsequently extracted from the pellet using a Maxwell RSC PureFood GMO and authentication kit (Promega) following the manufacturer’s instructions. Briefly, the fecal pellet was added to 1 mL of CTAB (cetyltrimethylammonium bromide) buffer and vortexed for 30 s, and then the sample was heated at 95°C for 5 min. After that, the samples were vortexed thoroughly with beads at maximum speed for 15 min. Then, 40 μL of proteinase K and 20 μL of RNase A were added to the sample, and the mixture was incubated at 70°C for 10 min. The supernatant was then obtained by centrifuging at 13,000 × *g* for 5 min and added to a Maxwell RSC machine for DNA extraction. The total extracted fecal DNA was used for metagenomics sequencing.

### VLP enrichment.

Virus-like particles (VLPs) were enriched by using a protocol described in a previous study (13). Two hundred milligrams of stool sample was added to 400 μL saline-magnesium buffer (0.1 M NaCl, 0.002% gelatin, 0.008 M MgSO_4_·H_2_O, 0.05 M Tris [pH 7.5]) and vortexed for 10 min. The sample then was centrifuged at 2,000 × *g*, and a suspension was obtained. To remove the bacterial cells and residual host cells, the suspension was further filtered by one 0.45-mm and two 0.22-mm filters. The cleared suspension was incubated with lysozyme (1 mg/mL at 37°C for 30 min) and chloroform (0.2× volume at room temperature [RT] for 10 min) in turn to degrade any remaining bacterial and host cell membranes. A DNase cocktail including 1 U Baseline Zero DNase (Epicenter) and 10 U Turbo DNase I (Ambion) was added to the sample, and the mixture was incubated at 65°C for 10 min to eliminate non-virus-protected DNA. VLPs were lysed (4% SDS plus 38 mg/mL proteinase K at 56°C for 20 min) and treated with CTAB (2.5% CTAB plus 0.5 M NaCl at 65°C for 10 min), and nucleic acid was extracted with phenol-chloroform (pH 8.0) (Invitrogen). The aqueous fraction was washed once with an equal volume of chloroform, purified, and concentrated on a column (DNA Clean & Concentrator 89-5; Zymo Research). VLP DNA was amplified for 2 h using Phi29 polymerase (GenomiPhi V2 kit; GE Healthcare) prior to sequencing. Four independent reactions were performed for each sample and amplified DNA was pooled to reduce.

### Metagenomics sequencing and analysis.

Qualified fecal DNA and VLP DNA were cut into fragments, and the sequencing libraries were prepared through the processes of end repairing, adding A to tails, purification, and PCR amplification. The fecal and VLP DNA libraries were sequenced on an Illumina NovaSeq 6000 system with a PE150 sequencing strategy by Novogene, Beijing, China, and yielded an average of 48 ± 5.3 million reads (12G data) and 25 ± 3.3 million reads (6G data) per sample, respectively. Raw sequence reads were filtered and quality trimmed using Trimmomatic v0.36 (35). Human reads were filtered out using Kneaddata (https://huttenhower.sph.harvard.edu/kneaddata/; reference database, GRCh38 p12) with the default argument to generate clean reads. Bacterial taxonomic and functional profiling were implemented in HUMAnN2 v0.11.1 (36). This workflow includes taxonomic identification by MetaPhlAn2 using clade-specific marker genes (37), annotation of species pangenomes through Bowtie2 (38) with the ChocoPhlAn database, a translated search of unmapped reads with DIAMOND (39) against the UniRef90 universal protein reference database (40), and pathway collection obtained from the generated gene list with reference to the MetaCyc database (41). The gene families and pathway abundance files of all samples were joined and normalized according to relative abundances for comparison between samples. ABRicate v.1.0.0 (https://github.com/tseemann/abricate) was used to screen for acquired antimicrobial resistance genes against CARD (42) databases (last updated November 2020). Genomic DNA was extracted from CRE isolates, and paired-end reads (150 bp) were generated on the Illumina HiSeq platforms and assembled with SPAdes v3.15.0.

### Viral classification, read assembly, and annotation.

Taxonomic profiles of viruses were first determined from the fecal DNA metagenomic data set, using Kraken2 v2.0.7-beta (43). The complete NCBI viral RefSeq database (accessed 18 January 2021) was downloaded (https://www.ncbi.nlm.nih.gov/refseq/). Each query was thereafter classified to a taxon with the highest total k-mer hits matched by pruning the general taxonomic trees affiliated with mapped genomes, as described previously (11). To improve the tentative classification of the gut virome, we additionally used assembly contigs for virome analysis. The quality-controlled reads were assembled into contigs using MEGAHIT (36) for each individual. To quantify contigs in each sample, the quality-controlled reads were mapped back to the contigs using Bowtie2 (37), and the number of mapped reads was calculated by processing SAM files using custom code. To remove differences in sequencing depth, reads per million total reads (RPM) were calculated for each contig. Assembled contigs from virome libraries with lengths greater than 3,000 bp and predicted as category 1 and 2 with VirSorter (22) were selected to predict open reading frames (ORFs) using Prodigal in “meta” mode (38). To annotate the predicted ORFs, the amino acid sequences of the ORFs were queried by Diamond (39) against the viral RefSeq protein (v84) with an E value of <10^−5^ and a bit score of >50. The viral RefSeq proteins with the closest homologies (E value of <10^−5^ and bit score of >50) were considered for each ORF, analogous to a previously reported method (44).

### Animal experiment.

C57BL/6J male mice were used at 6 to 8 weeks of age and were randomly assigned to experimental and control groups. In all experiments, age- and gender-matched mice were used. All mice were kept at a strict 24-h light-dark cycle, with lights on from 6 a.m. to 6 p.m. For antibiotic treatment, mice were given vancomycin (0.125 g)-neomycin (0.25 g)-metronidazole (0.25 g)-ampicillin (0.25 g) (combined in 250 mL water) in their drinking water for 2 weeks as previously described (45). On the day of FMT, a fresh FMT was prepared by harvesting stools from normal healthy mice. The stool pellets were then suspended in 100 μL sterile PBS, and mice were subsequently given a 100-μL suspension by oral gavage. Fecal virome fraction transplantation (FVT) was carried out with a VLP preparation. A stool pellet from the untreated healthy mice were suspended in 300 μL sterile PBS and centrifuged at 2,500 × *g* for 10 min. Then, bacteria were removed in the VLP-containing supernatant using a 0.45-μm filter, followed by a 0.22-μm filter. A 100-kDa centrifugal filter was used at 3,220 × *g* for 5 min to capture VLPs in the fecal filtrate and then washed 3 times with PBS under the same conditions. Afterward, VLPs on the filter were suspended in 100 μL PBS, and mice received a 100-μL suspension by oral gavage. Control mice received the same volume of PBS as their FMT/FVT group counterparts. All experimental procedures were approved by the Animal Ethics Committee of the Chinese University of Hong Kong.

### Culture-based analyses and molecular identification of carbapenemase.

Stool samples (200 mg) were plated, transferred into 1 mL phosphate-buffered saline buffer, and agitated to release the microorganisms. An inoculum volume of 100 μL was directly plated onto chromID Carba agar (bioMérieux, France), which consists of a nutrient base combining different peptones, three chromogenic substrates enabling the detection of activities of specific metabolic enzymes for Escherichia coli, Klebsiella/Enterobacter/*Serratia*/*Citrobacter*, and *Proteeae*, and a proprietary mixture of antibiotics favoring the selective growth of carbapenemase-producing *Enterobacteriaceae*. Species identification was further performed using matrix-assisted laser desorption ionization–time-of-flight mass spectrometry (MALDI-TOF MS) (Bruker Daltonics, Bremen, Germany) (Table S1). Carbapenemase genes (*bla*_KPC_, *bla*_VIM_, *bla*_IMP_, *bla*_NDM_, and *bla*_OXA-48-like_) were detected by multiplex PCR (46–48). Antibiotic susceptibility test results for CRE isolates are shown in Table S2. The distribution of the genetic determinants of resistance detected by whole-genome sequencing (WGS) in each CRE isolate is shown in Fig. S5.

10.1128/msystems.01510-21.1 TABLE S1 CRE isolates and their detected carbapenemase genes. Download Table S1, DOCX file, 0.01 MB. Copyright © 2022 Liu et al. 2022 Liu et al. https://creativecommons.org/licenses/by/4.0/ This content is distributed under the terms of the Creative Commons Attribution 4.0 International license.

10.1128/msystems.01510-21.2 TABLE S2 Antibiotic susceptibility test results for CRE isolates. Download Table S2, DOCX file, 0.01 MB. Copyright © 2022 Liu et al. 2022 Liu et al. https://creativecommons.org/licenses/by/4.0/ This content is distributed under the terms of the Creative Commons Attribution 4.0 International license.

10.1128/msystems.01510-21.7 FIG S5 Distribution of antimicrobial resistance (AMR) genes detected by whole-genome sequencing in each CRE isolate. Download FIG S5, JPG file, 0.1 MB. Copyright © 2022 Liu et al. 2022 Liu et al. https://creativecommons.org/licenses/by/4.0/ This content is distributed under the terms of the Creative Commons Attribution 4.0 International license.

### Quantification of abundance of Klebsiella pneumoniae.

For quantification of K. pneumoniae loads in human stools, quantitative PCR amplifications were performed using the K. pneumoniae-specific primers (49) K. pneumoniae-F (5′-ATTTGAAGAGGTTGCAAACGAT-3′) and K. pneumoniae-R (5′-TTCACTCTGAAGTTTTCTTGTGTTC-3′) and the universal 16S rRNA primers 16s-1114_F (5′-CGGCAACGAGCGCAACCC-3′) and 16s-1275_R (5′-CCATTGTAGCACGTGTGTAGCC-3′) (50). The primers were validated by determining qPCR efficiency using isolated bacterial DNA from a reference K. pneumoniae strain. Replicates were averaged, and bacterial load was quantified using the 2^−ΔΔ^*^CT^* method per the following formula: 2^−[(^*^CT^* ^of experimental sample –^*^CT^* ^of control bacteria 16S) – (^*^CT^* ^of control sample –^*^CT^* ^of control bacteria 16S)]^. The assay was performed using Power SYBR green PCR master mix (TaKaRa BioMedicals, Japan) with the StepOne real-time PCR system.

### Quantification and statistics.

The abundance data for bacteria and viruses were imported into R 3.3.5. Diversity and rarefaction calculations were performed using the Phyloseq package. Alpha diversity was analyzed by calculating the Shannon index (a proxy for diversity, taking into account both richness and evenness) and Chao1 (a proxy for community richness). Principal-coordinate analysis (PCoA) based on the Bray-Curtis dissimilarity matrix of the microbial community structure was calculated with the vegan R package. Linear discriminant analysis (LDA) effect size (LEfSe) (37) was performed using the online tool available at http://huttenhower.sph.harvard.edu/galaxy/. LDA identifies taxa based on their contribution to the overall observed differences between groups, that is, taxa that are significantly increased in abundance. Statistical significance was verified through analysis of variance with *post hoc* multiple-comparison testing between groups or the nonparametric Wilcoxon rank-sum analysis, as reported above. Differences in bacterial and viral abundance were calculated using LEfSe (37). Plots were generated with RStudio (using ggplot2 R package) and edited with Illustrator CC (V21.0.0). Statistical analysis was performed using RStudio.

### Data availability.

Raw data have been deposited in a BioProject in the NCBI Sequence Read Archive: PRJNA556087. Data are available on reasonable request.

## References

[1] van Duin D, Doi Y. 2017. The global epidemiology of carbapenemase-producing Enterobacteriaceae. Virulence 8:460–469. doi:10.1080/21505594.2016.1222343. 27593176 PMC5477705

[2] Rubinstein E, Keynan Y. 2013. Vancomycin-resistant enterococci. Crit Care Clin 29:841–852. doi:10.1016/j.ccc.2013.06.006. 24094380

[3] Saha S, Tariq R, Tosh P, Pardi D, Khanna S. 2019. Faecal microbiota transplantation for eradicating carriage of multidrug-resistant organisms: a systematic review. Clin Microbiol Infect 25:958–963. doi:10.1016/j.cmi.2019.04.006. 30986562

[4] Venkatesan P. 2021. European Congress of Clinical Microbiology & Infectious Diseases 2021. Lancet Microbe 2:e427–e428. doi:10.1016/S2666-5247(21)00213-5. 35544148

[5] Oostdijk EA, de Smet AMG, Kesecioglu J, Bonten MJ, Dutch SOD-SDD Trialists Group. 2012. Decontamination of cephalosporin-resistant Enterobacteriaceae during selective digestive tract decontamination in intensive care units. J Antimicrob Chemother 67:2250–2253. doi:10.1093/jac/dks187. 22643189

[6] van Nood E, Vrieze A, Nieuwdorp M, Fuentes S, Zoetendal EG, de Vos WM, Visser CE, Kuijper EJ, Bartelsman JFWM, Tijssen JGP, Speelman P, Dijkgraaf MGW, Keller JJ. 2013. Duodenal infusion of donor feces for recurrent Clostridium difficile. N Engl J Med 368:407–415. doi:10.1056/NEJMoa1205037. 23323867

[7] Manges AR, Steiner TS, Wright AJ. 2016. Fecal microbiota transplantation for the intestinal decolonization of extensively antimicrobial-resistant opportunistic pathogens: a review. Infect Dis (Lond) 48:587–592. doi:10.1080/23744235.2016.1177199. 27194400

[8] Gopalsamy SN, Sherman A, Woodworth MH, Lutgring JD, Kraft CS. 2018. Fecal microbiota transplant for multidrug-resistant organism decolonization administered during septic shock. Infect Control Hosp Epidemiol 39:490–492. doi:10.1017/ice.2017.300. 29343312 PMC5996996

[9] Davido B, Batista R, Michelon H, Lepainteur M, Bouchand F, Lepeule R, Salomon J, Vittecoq D, Duran C, Escaut L, Sobhani I, Paul M, Lawrence C, Perronne C, Chast F, Dinh A. 2017. Is faecal microbiota transplantation an option to eradicate highly drug-resistant enteric bacteria carriage? J Hosp Infect 95:433–437. doi:10.1016/j.jhin.2017.02.001. 28237504

[10] Dinh A, Fessi H, Duran C, Batista R, Michelon H, Bouchand F, Lepeule R, Vittecoq D, Escaut L, Sobhani I, Lawrence C, Chast F, Ronco P, Davido B. 2018. Clearance of carbapenem-resistant Enterobacteriaceae vs vancomycin-resistant enterococci carriage after faecal microbiota transplant: a prospective comparative study. J Hosp Infect 99:481–486. doi:10.1016/j.jhin.2018.02.018. 29477634

[11] Zhang F, Zuo T, Yeoh YK, Cheng FWT, Liu Q, Tang W, Cheung KCY, Yang K, Cheung CP, Mo CC, Hui M, Chan FKL, Li C-K, Chan PKS, Ng SC. 2021. Longitudinal dynamics of gut bacteriome, mycobiome and virome after fecal microbiota transplantation in graft-versus-host disease. Nat Commun 12:65. doi:10.1038/s41467-020-20240-x. 33397897 PMC7782528

[12] Lee J-J, Yong D, Suk KT, Kim DJ, Woo H-J, Lee SS, Kim B-S. 2021. Alteration of gut microbiota in carbapenem-resistant Enterobacteriaceae carriers during fecal microbiota transplantation according to decolonization periods. Microorganisms 9:352. doi:10.3390/microorganisms9020352. 33578974 PMC7916679

[13] Zuo T, Wong SH, Lam K, Lui R, Cheung K, Tang W, Ching JYL, Chan PKS, Chan MCW, Wu JCY, Chan FKL, Yu J, Sung JJY, Ng SC. 2018. Bacteriophage transfer during faecal microbiota transplantation in Clostridium difficile infection is associated with treatment outcome. Gut 67:634–643. doi:10.1136/gutjnl-2017-313952. 28539351 PMC5868238

[14] Zuo T, Wong SH, Cheung CP, Lam K, Lui R, Cheung K, Zhang F, Tang W, Ching JYL, Wu JCY, Chan PKS, Sung JJY, Yu J, Chan FKL, Ng SC. 2018. Gut fungal dysbiosis correlates with reduced efficacy of fecal microbiota transplantation in clostridium difficile infection. Nat Commun 9:3663. doi:10.1038/s41467-018-06103-6. 30202057 PMC6131390

[15] Knights D, Kuczynski J, Charlson ES, Zaneveld J, Mozer MC, Collman RG, Bushman FD, Knight R, Kelley ST. 2011. Bayesian community-wide culture-independent microbial source tracking. Nat Methods 8:761–763. doi:10.1038/nmeth.1650. 21765408 PMC3791591

[16] Drulis-Kawa Z, Mackiewicz P, Kęsik-Szeloch A, Maciaszczyk-Dziubinska E, Weber-Dąbrowska B, Dorotkiewicz-Jach A, Augustyniak D, Majkowska-Skrobek G, Bocer T, Empel J, Kropinski AM. 2011. Isolation and characterisation of KP34—a novel φKMV-like bacteriophage for Klebsiella pneumoniae. Appl Microbiol Biotechnol 90:1333–1345. doi:10.1007/s00253-011-3149-y. 21327407 PMC3082699

[17] Stern A, Mick E, Tirosh I, Sagy O, Sorek R. 2012. CRISPR targeting reveals a reservoir of common phages associated with the human gut microbiome. Genome Res 22:1985–1994. doi:10.1101/gr.138297.112. 22732228 PMC3460193

[18] Roux S, Enault F, Hurwitz BL, Sullivan MB. 2015. VirSorter: mining viral signal from microbial genomic data. PeerJ 3:e985. doi:10.7717/peerj.985. 26038737 PMC4451026

[19] Buffie CG, Pamer EG. 2013. Microbiota-mediated colonization resistance against intestinal pathogens. Nat Rev Immunol 13:790–801. doi:10.1038/nri3535. 24096337 PMC4194195

[20] Khoruts A, Sadowsky MJ. 2016. Understanding the mechanisms of faecal microbiota transplantation. Nat Rev Gastroenterol Hepatol 13:508–516. doi:10.1038/nrgastro.2016.98. 27329806 PMC5909819

[21] Lenski RE, Riley MA. 2002. Chemical warfare from an ecological perspective. Proc Natl Acad Sci USA 99:556–558. doi:10.1073/pnas.022641999. 11805313 PMC117343

[22] Bar-Yoseph H, Carasso S, Shklar S, Korytny A, Even Dar R, Daoud H, Nassar R, Maharshak N, Hussein K, Geffen Y, Chowers Y, Geva-Zatorsky N, Paul M. 2021. Oral capsulized fecal microbiota transplantation for eradication of carbapenemase-producing Enterobacteriaceae colonization with a metagenomic perspective. Clin Infect Dis 73:e166–e175. doi:10.1093/cid/ciaa737. 32511695

[23] Wilson BC, Vatanen T, Cutfield WS, O'Sullivan JM. 2019. The super-donor phenomenon in fecal microbiota transplantation. Front Cell Infect Microbiol 9:2. doi:10.3389/fcimb.2019.00002. 30719428 PMC6348388

[24] Gopalakrishnan V, Spencer CN, Nezi L, Reuben A, Andrews MC, Karpinets TV, Prieto PA, Vicente D, Hoffman K, Wei SC, Cogdill AP, Zhao L, Hudgens CW, Hutchinson DS, Manzo T, Petaccia de Macedo M, Cotechini T, Kumar T, Chen WS, Reddy SM, Szczepaniak Sloane R, Galloway-Pena J, Jiang H, Chen PL, Shpall EJ, Rezvani K, Alousi AM, Chemaly RF, Shelburne S, Vence LM, Okhuysen PC, Jensen VB, Swennes AG, McAllister F, Marcelo Riquelme Sanchez E, Zhang Y, Le Chatelier E, Zitvogel L, Pons N, Austin-Breneman JL, Haydu LE, Burton EM, Gardner JM, Sirmans E, Hu J, Lazar AJ, Tsujikawa T, Diab A, Tawbi H, Glitza IC, et al. 2018. Gut microbiome modulates response to anti–PD-1 immunotherapy in melanoma patients. Science 359:97–103. doi:10.1126/science.aan4236. 29097493 PMC5827966

[25] Zechner EL. 2017. Inflammatory disease caused by intestinal pathobionts. Curr Opin Microbiol 35:64–69. doi:10.1016/j.mib.2017.01.011. 28189956

[26] Danne C, Rolhion N, Sokol H. 2021. Recipient factors in faecal microbiota transplantation: one stool does not fit all. Nat Rev Gastroenterol Hepatol 18:503–511. doi:10.1038/s41575-021-00441-5. 33907321

[27] Reyes A, Haynes M, Hanson N, Angly FE, Heath AC, Rohwer F, Gordon JI. 2010. Viruses in the faecal microbiota of monozygotic twins and their mothers. Nature 466:334–338. doi:10.1038/nature09199. 20631792 PMC2919852

[28] Minot S, Sinha R, Chen J, Li H, Keilbaugh SA, Wu GD, Lewis JD, Bushman FD. 2011. The human gut virome: inter-individual variation and dynamic response to diet. Genome Res 21:1616–1625. doi:10.1101/gr.122705.111. 21880779 PMC3202279

[29] Rodriguez-Valera F, Martin-Cuadrado A-B, Rodriguez-Brito B, Pašić L, Thingstad TF, Rohwer F, Mira A. 2009. Explaining microbial population genomics through phage predation. Nat Rev Microbiol 7:828–836. doi:10.1038/nrmicro2235. 19834481

[30] Rios AC, Moutinho CG, Pinto FC, Del Fiol FS, Jozala A, Chaud MV, Vila MM, Teixeira JA, Balcão VM. 2016. Alternatives to overcoming bacterial resistances: state-of-the-art. Microbiol Res 191:51–80. doi:10.1016/j.micres.2016.04.008. 27524653

[31] Ogilvie LA, Jones BV. 2015. The human gut virome: a multifaceted majority. Front Microbiol 6:918. doi:10.3389/fmicb.2015.00918. 26441861 PMC4566309

[32] Labrie SJ, Samson JE, Moineau S. 2010. Bacteriophage resistance mechanisms. Nat Rev Microbiol 8:317–327. doi:10.1038/nrmicro2315. 20348932

[33] Kortright KE, Chan BK, Koff JL, Turner PE. 2019. Phage therapy: a renewed approach to combat antibiotic-resistant bacteria. Cell Host Microbe 25:219–232. doi:10.1016/j.chom.2019.01.014. 30763536

[34] Simmonds P, Adams MJ, Benkő M, Breitbart M, Brister JR, Carstens EB, Davison AJ, Delwart E, Gorbalenya AE, Harrach B, Hull R, King AMQ, Koonin EV, Krupovic M, Kuhn JH, Lefkowitz EJ, Nibert ML, Orton R, Roossinck MJ, Sabanadzovic S, Sullivan MB, Suttle CA, Tesh RB, van der Vlugt RA, Varsani A, Zerbini FM. 2017. Consensus statement: virus taxonomy in the age of metagenomics. Nat Rev Microbiol 15:161–168. doi:10.1038/nrmicro.2016.177. 28134265

[35] Bolger AM, Lohse M, Usadel B. 2014. Trimmomatic: a flexible trimmer for Illumina sequence data. Bioinformatics 30:2114–2120. doi:10.1093/bioinformatics/btu170. 24695404 PMC4103590

[36] Franzosa EA, McIver LJ, Rahnavard G, Thompson LR, Schirmer M, Weingart G, Lipson KS, Knight R, Caporaso JG, Segata N, Huttenhower C. 2018. Species-level functional profiling of metagenomes and metatranscriptomes. Nat Methods 15:962–968. doi:10.1038/s41592-018-0176-y. 30377376 PMC6235447

[37] Segata N, Izard J, Waldron L, Gevers D, Miropolsky L, Garrett WS, Huttenhower C. 2011. Metagenomic biomarker discovery and explanation. Genome Biol 12:R60. doi:10.1186/gb-2011-12-6-r60. 21702898 PMC3218848

[38] Langmead B, Salzberg SL. 2012. Fast gapped-read alignment with Bowtie 2. Nat Methods 9:357–359. doi:10.1038/nmeth.1923. 22388286 PMC3322381

[39] Buchfink B, Xie C, Huson DH. 2015. Fast and sensitive protein alignment using DIAMOND. Nat Methods 12:59–60. doi:10.1038/nmeth.3176. 25402007

[40] Suzek BE, Wang Y, Huang H, McGarvey PB, Wu CH, Consortium U, the UniProt Consortium. 2015. UniRef clusters: a comprehensive and scalable alternative for improving sequence similarity searches. Bioinformatics 31:926–932. doi:10.1093/bioinformatics/btu739. 25398609 PMC4375400

[41] Caspi R, Altman T, Billington R, Dreher K, Foerster H, Fulcher CA, Holland TA, Keseler IM, Kothari A, Kubo A, Krummenacker M, Latendresse M, Mueller LA, Ong Q, Paley S, Subhraveti P, Weaver DS, Weerasinghe D, Zhang P, Karp PD. 2014. The MetaCyc database of metabolic pathways and enzymes and the BioCyc collection of pathway/genome databases. Nucleic Acids Res 42:D459–D471. doi:10.1093/nar/gkt1103. 24225315 PMC3964957

[42] McArthur AG, Waglechner N, Nizam F, Yan A, Azad MA, Baylay AJ, Bhullar K, Canova MJ, De Pascale G, Ejim L, Kalan L, King AM, Koteva K, Morar M, Mulvey MR, O'Brien JS, Pawlowski AC, Piddock LJV, Spanogiannopoulos P, Sutherland AD, Tang I, Taylor PL, Thaker M, Wang W, Yan M, Yu T, Wright GD. 2013. The comprehensive antibiotic resistance database. Antimicrob Agents Chemother 57:3348–3357. doi:10.1128/AAC.00419-13. 23650175 PMC3697360

[43] Wood DE, Salzberg SL. 2014. Kraken: ultrafast metagenomic sequence classification using exact alignments. Genome Biol 15:R46. doi:10.1186/gb-2014-15-3-r46. 24580807 PMC4053813

[44] Kang D-W, Adams JB, Gregory AC, Borody T, Chittick L, Fasano A, Khoruts A, Geis E, Maldonado J, McDonough-Means S, Pollard EL, Roux S, Sadowsky MJ, Lipson KS, Sullivan MB, Caporaso JG, Krajmalnik-Brown R. 2017. Microbiota transfer therapy alters gut ecosystem and improves gastrointestinal and autism symptoms: an open-label study. Microbiome 5:10. doi:10.1186/s40168-016-0225-7. 28122648 PMC5264285

[45] Croswell A, Amir E, Teggatz P, Barman M, Salzman NH. 2009. Prolonged impact of antibiotics on intestinal microbial ecology and susceptibility to enteric Salmonella infection. Infect Immun 77:2741–2753. doi:10.1128/IAI.00006-09. 19380465 PMC2708550

[46] Tsakris A, Kristo I, Poulou A, Themeli-Digalaki K, Ikonomidis A, Petropoulou D, Pournaras S, Sofianou D. 2009. Evaluation of boronic acid disk tests for differentiating KPC-possessing Klebsiella pneumoniae isolates in the clinical laboratory. J Clin Microbiol 47:362–367. doi:10.1128/JCM.01922-08. 19073868 PMC2643660

[47] Perry JD, Naqvi SH, Mirza IA, Alizai SA, Hussain A, Ghirardi S, Orenga S, Wilkinson K, Woodford N, Zhang J, Livermore DM, Abbasi SA, Raza MW. 2011. Prevalence of faecal carriage of Enterobacteriaceae with NDM-1 carbapenemase at military hospitals in Pakistan, and evaluation of two chromogenic media. J Antimicrob Chemother 66:2288–2294. doi:10.1093/jac/dkr299. 21788293

[48] Poirel L, Héritier C, Tolün V, Nordmann P. 2004. Emergence of oxacillinase-mediated resistance to imipenem in Klebsiella pneumoniae. Antimicrob Agents Chemother 48:15–22. doi:10.1128/AAC.48.1.15-22.2004. 14693513 PMC310167

[49] Mahmudunnabi G, Majlish ANK, Momtaz F, Foysal MJ, Rahman MM, Islam K. 2018. Molecular detection and PCR-RFLP analysis using Pst1 and Alu1 of multidrug resistant Klebsiella pneumoniae causing urinary tract infection in women in the eastern part of Bangladesh. J Genet Eng Biotechnol 16:77–82. doi:10.1016/j.jgeb.2017.12.004. 30647708 PMC6296566

[50] Ruff WE, Dehner C, Kim WJ, Pagovich O, Aguiar CL, Yu AT, Roth AS, Vieira SM, Kriegel C, Adeniyi O, Mulla MJ, Abrahams VM, Kwok WW, Nussinov R, Erkan D, Goodman AL, Kriegel MA. 2019. Pathogenic autoreactive T and B cells cross-react with mimotopes expressed by a common human gut commensal to trigger autoimmunity. Cell Host Microbe 26:100–113.E8. doi:10.1016/j.chom.2019.05.003. 31227334 PMC8194364

