# Supplementary figures and images for: Longitudinal Evaluation of Gut Bacteriomes and Viromes after Fecal Microbiota Transplantation for Eradication of Carbapenem-Resistant Enterobacteriaceae
Source: mSystems. 2022 Jun 1;7(3):e01510-21. doi: 10.1128/msystems.01510-21 (PMC9239097; doi:10.1128/msystems.01510-21)

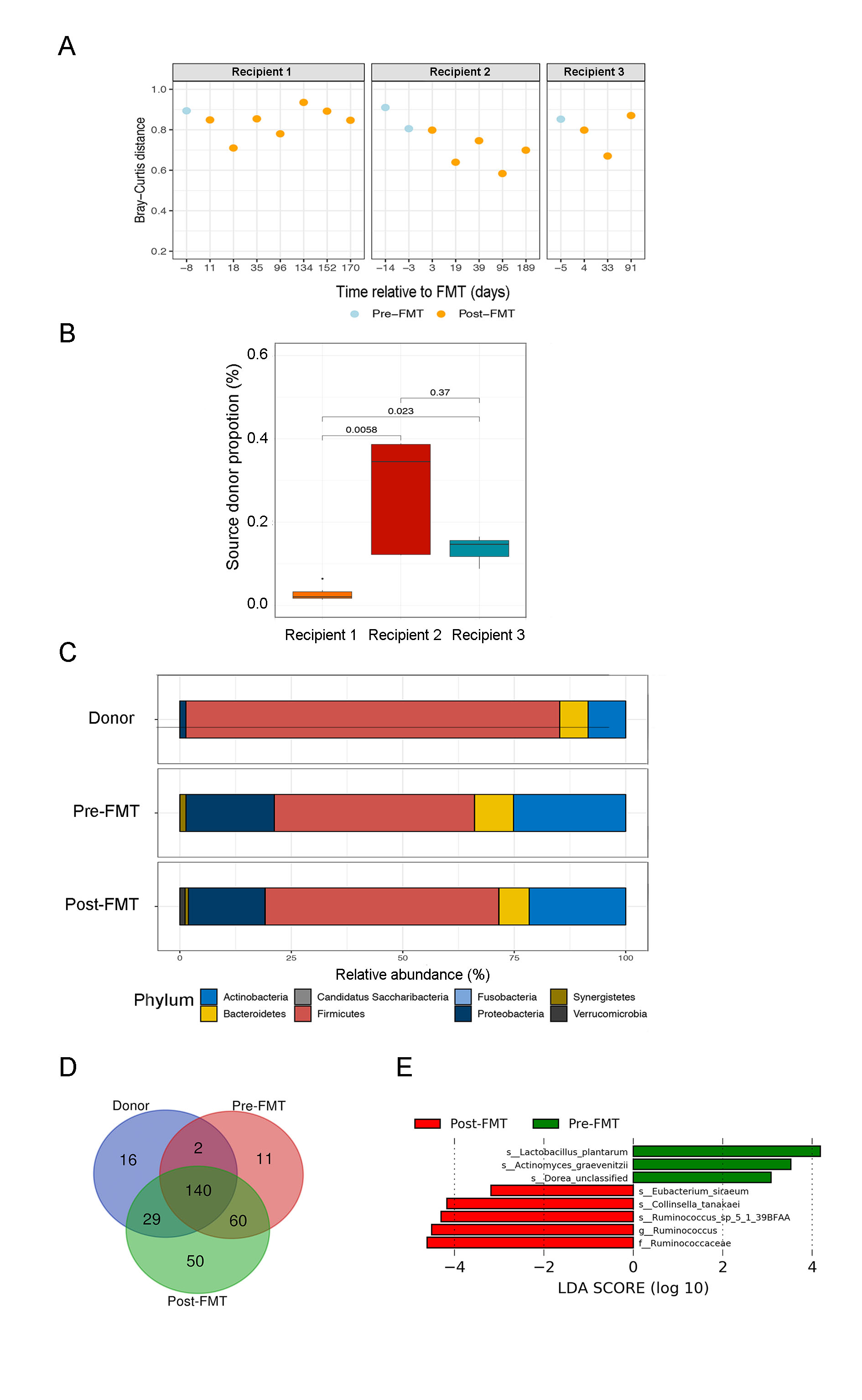

Supplement: FIG S1 [file msystems.01510-21-s0003.jpg]

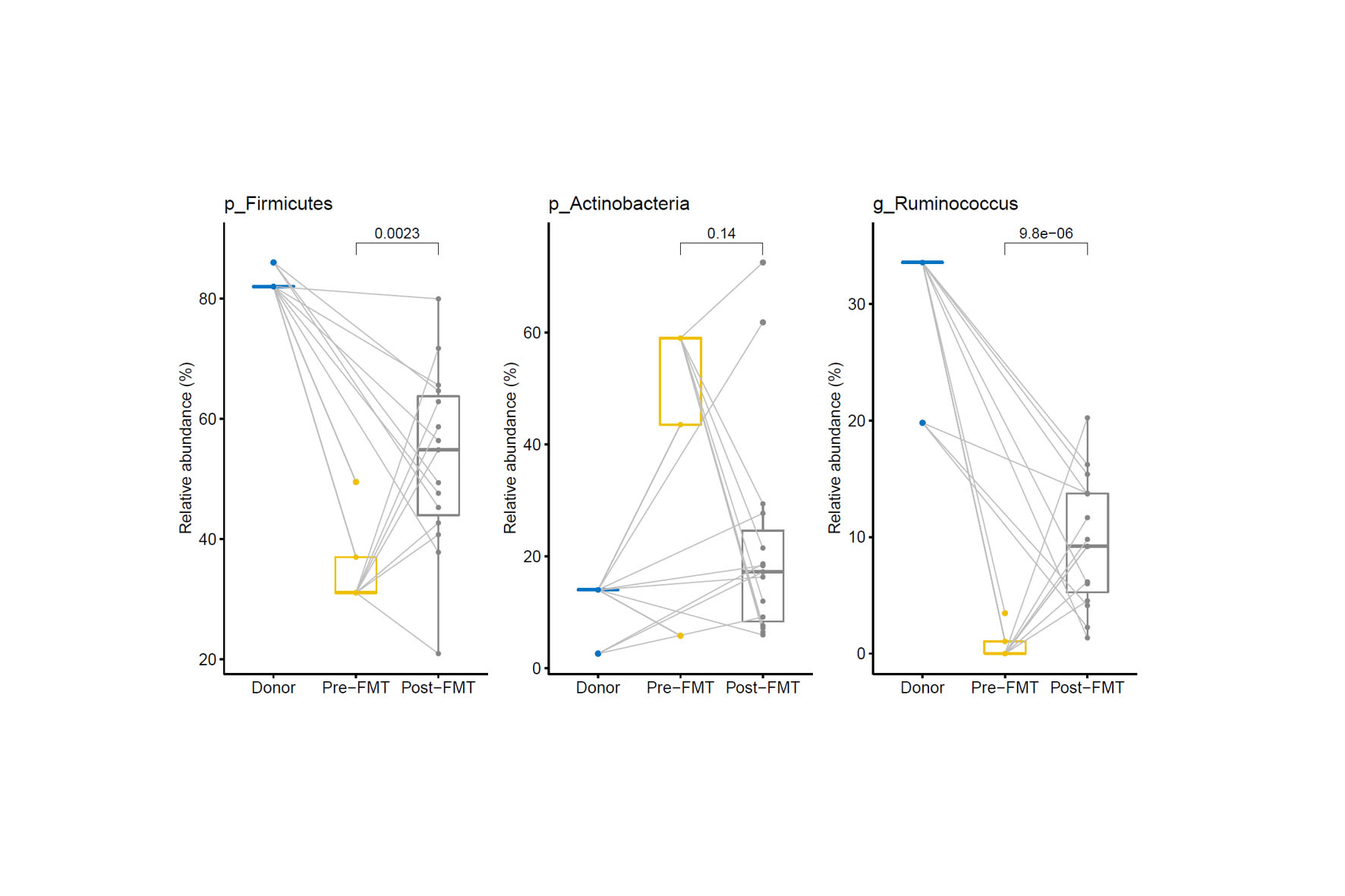

Supplement: FIG S2 [file msystems.01510-21-s0004.jpg]

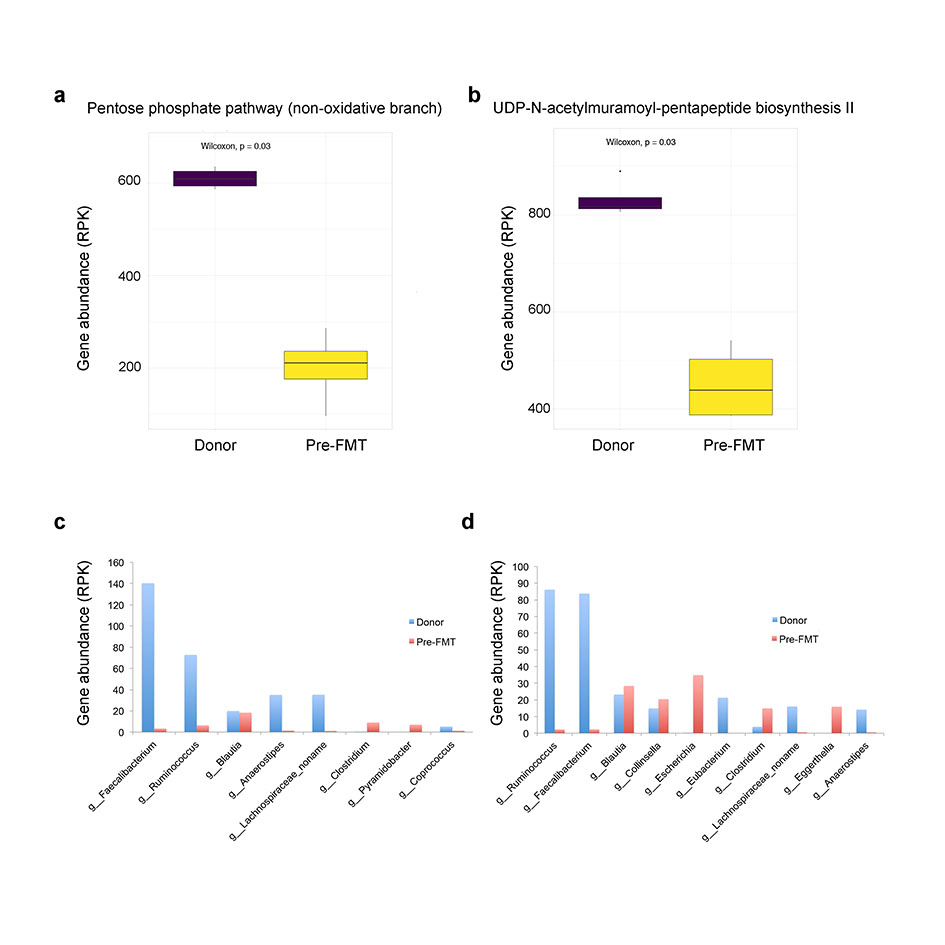

Supplement: FIG S3 [file msystems.01510-21-s0005.jpg]

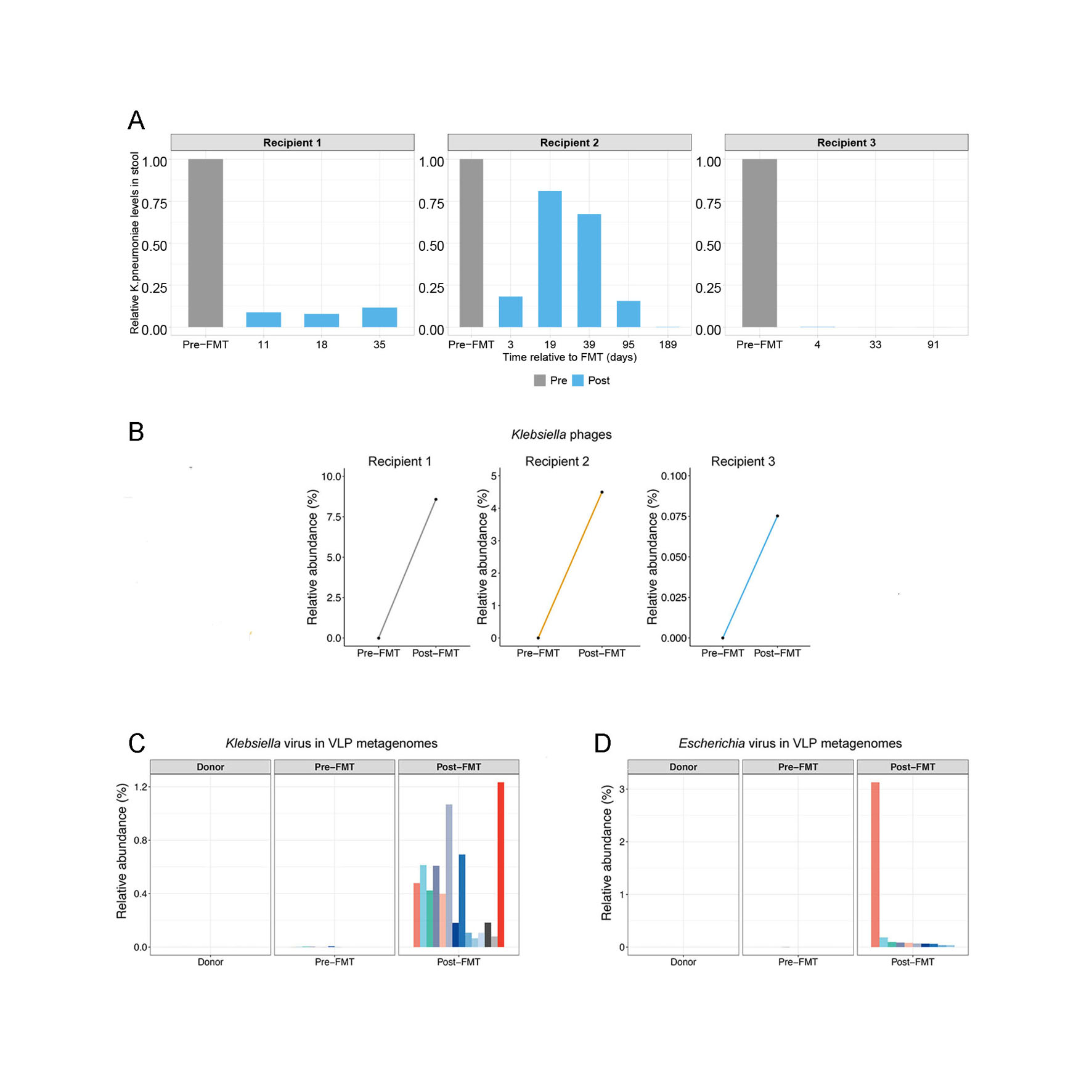

Supplement: FIG S4 [file msystems.01510-21-s0006.jpg]

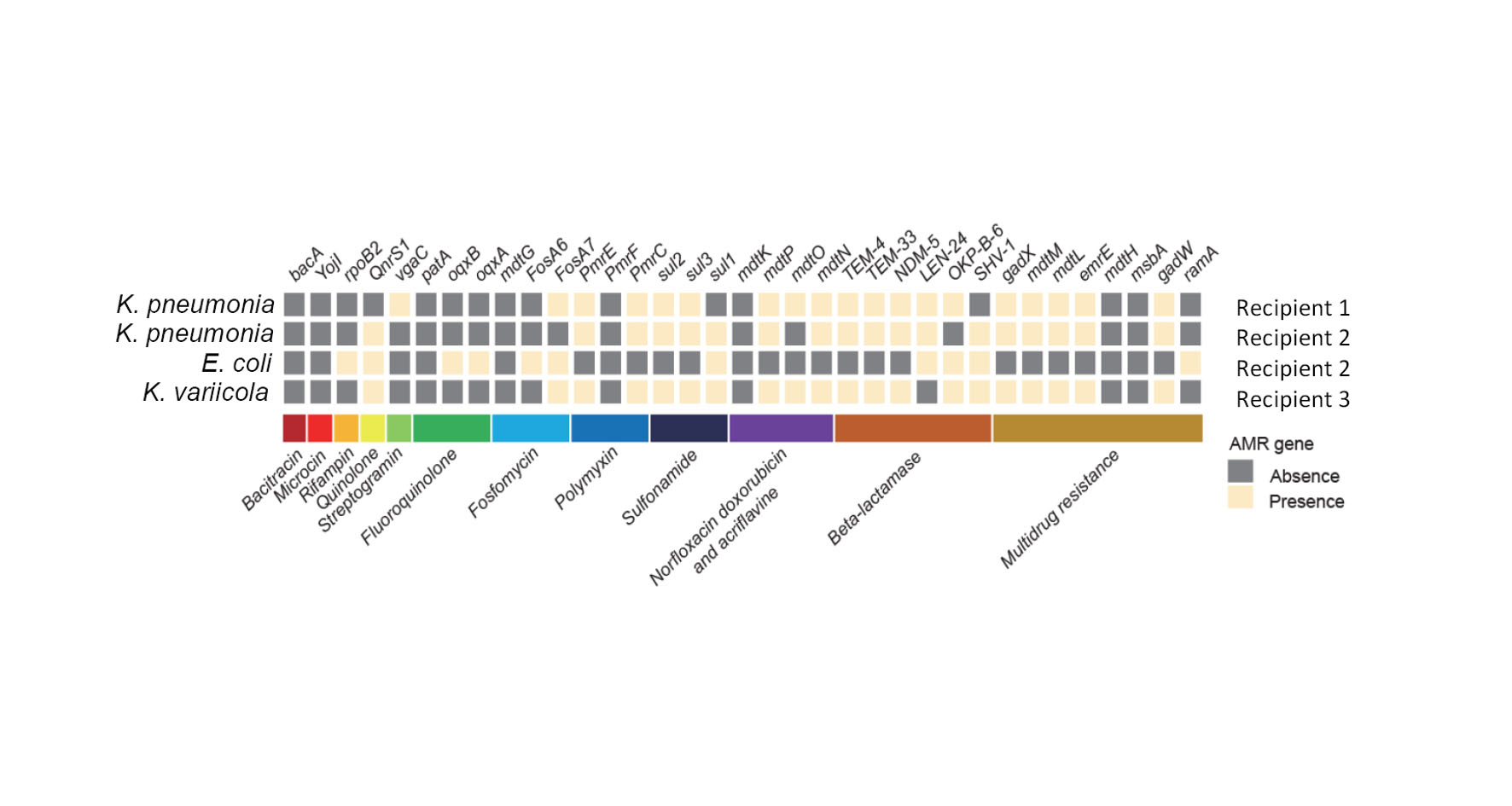

Supplement: FIG S5 [file msystems.01510-21-s0007.jpg]
