# Supplementary material for: Longitudinal Evaluation of Gut Bacteriomes and Viromes after Fecal Microbiota Transplantation for Eradication of Carbapenem-Resistant Enterobacteriaceae
Source: mSystems. 2022 Jun 1;7(3):e01510-21. doi: 10.1128/msystems.01510-21 (PMC9239097; doi:10.1128/msystems.01510-21)
Supplement: TABLE S1 [file msystems.01510-21-s0001.docx]

**Supplementary Table 1 CRE isolates and their detected carbapenemase genes**

| Recipient | Species | Carbapenemase gene type |
| --- | --- | --- |
| 1 | *K. pneumonia* | OXA-181 |
| 2 | *K. pneumonia* | NDM-1 |
|  | *E. coli* | NDM-5 |
| 3 | *K. variicola* | NDM |
