# Supplementary material for: Longitudinal Evaluation of Gut Bacteriomes and Viromes after Fecal Microbiota Transplantation for Eradication of Carbapenem-Resistant Enterobacteriaceae
Source: mSystems. 2022 Jun 1;7(3):e01510-21. doi: 10.1128/msystems.01510-21 (PMC9239097; doi:10.1128/msystems.01510-21)
Supplement: TABLE S2 [file msystems.01510-21-s0002.docx]

**Supplementary Table 2 Antibiotics susceptibility test results of CRE isolates**

| Antibiotics | Recipient 1 | | Recipient 2 | | Recipient 3 | | |
| --- | --- | --- | --- | --- | --- | --- | --- |
|  | Specimen:  Rectal swap | Specimen：Stool | Specimen：  Stool | Specimen：Stool | | Specimen:  Rectal Swab | Specimen：Stool |
|  | Culture: Klebsiella species | Culture:  Klebsiella species | Culture:  Klebsiella species | Culture:  Escherichia coli | | Culture: Klebsiella species | Culture:  Klebsiella species |
| Amikacin | NS | NS | S | S | | R | R |
| Amoxycillin/clavulanate | R | R | R | R | | R | R |
| Cefepime | NS | NS | I | R | | R | R |
| Cefoperazone/sulbactam | I | I | I | R | | R | R |
| Cefotaxime | NS | I | R | R | | R | R |
| Cefotaxime (parenteral) | S | S | R | R | | R | R |
| Ciprofloxacin | I | I | S | R | | R | R |
| Ertapenem | I | S | I | R | | R | R |
| Gentamicin | S | S | R | R | | R | R |
| Imipenem | I | S | I | R | | I | R |
| Piperacillin/tazobactam | R | R | R | R | | R | R |

S:Sensitive I:Intermediate R:Resistant

MR:Moderate SD:Susceptible-dose dependent SU:Susceptible NS：non-susceptible
